# New insights into genome assembly at the chromosome‐level of *Prunus tomentosa* in evolution and cold tolerance

**DOI:** 10.1002/imt2.70016

**Published:** 2025-03-20

**Authors:** Songtao Jiu, Muhammad Aamir Manzoor, Zhengxin Lv, Baozheng Chen, Shaoqin Shen, Yan Xu, Moyang Liu, Chengwei Li, Xunju Liu, Yanhong Fu, Qijing Zhang, Ruie Liu, Xinyu Zhang, Shiping Wang, Xiaoming Song, Yang Dong, Caixi Zhang

**Affiliations:** ^1^ Department of Plant Science, School of Agriculture and Biology Shanghai Jiao Tong University Shanghai P. R. China; ^2^ Province Key Laboratory, Biological Big Data College Yunnan Agricultural University Kunming P. R. China; ^3^ College of Life Sciences North China University of Science and Technology Tangshan P. R. China; ^4^ Department of Molecular, Cellular & Developmental Biology, College of Arts and Sciences University of Colorado Boulder Boulder Colorado USA; ^5^ Liaoning Institute of Pomology, Liaoning Academy of Agricultural Sciences Yingkou P. R. China

## Abstract

This study assembled a high‐quality chromosome‐level genome of *Prunus tomentosa*, offering a vital resource for elucidating its genetic architecture, evolutionary relationships, and facilitating genome‐assisted breeding efforts. Multi‐omics integration revealed *PtIMP3* and *PtMIOX1L* as key factors in cold tolerance of *P. tomentosa*. *PtIMP3* drives the conversion of glucose‐6‐phosphate to *myo*‐inositol, while *PtMIOX1L* catalyzes *myo*‐inositol to d‐glucuronic acid. Specifically, the high expression abundance of *PtIMP3* and low expression abundance of *PtMIOX1L* resulted in high endogenous inositol levels in *P. tomentosa*. The application of *myo*‐inositol enhanced the cold tolerance of cherry rootstocks by modulating reactive oxygen species concentrations and maintaining a stable relative water content. This finding supports the superior performance of *P. tomentosa* in adapting to extreme low‐temperatures environmental conditions. These insights advance strategies for improving cold tolerance in horticultural crops, bridging fundamental research with practical applications in developing climate‐resilient crops.

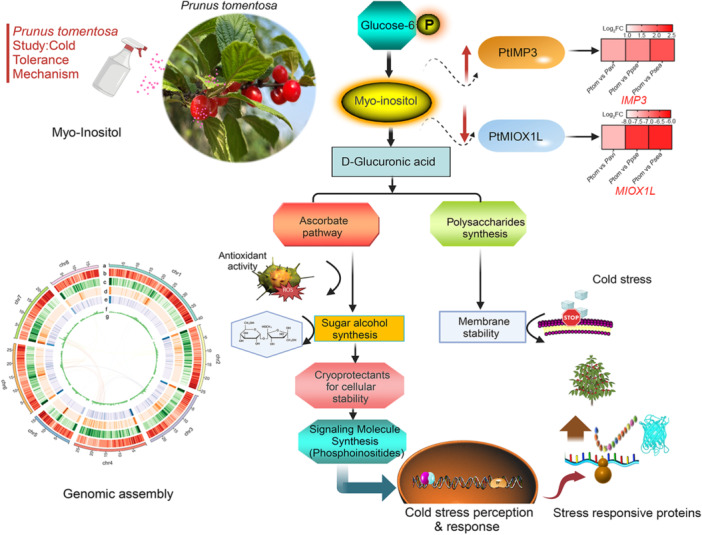

To the Editor,

The family Rosaceae, comprising over 100 genera and approximately 3000 species, plays a crucial role in horticulture and agricultural production globally [1]. *Prunus L*., encompassing over 200 species of shrubs and trees, is a significant component of Northern Hemisphere forests and desert ecosystems [2]. The classification of genus *Prunus* L., previously subdivided into six subgenera (*Amygdalus*, *Cerasus*, *Laurocerasus*, *Microcerasus*, *Padus*, and *Prunophora*) [3, 4], has been controversial, partly owing to the ease of interspecific hybridization [5]. *Prunus tomentosa* Thunb., commonly known as Nanking cherry, has long been classified within the subgenus *Microcerasus* [6]. However, studies on high‐quality chromosome‐scale genomes and phylogenetic analyses of *P. tomentosa* are lacking, rendering its genetic background relatively unknown.

*P. tomentosa* has been a component of the Chinese diet for over 2000 years, noted for its rich content of vitamins (B1, B2, C, D, and E) and other antioxidant compounds, such as carotene and niacin [7, 8]. It features dark green oval leaves, approximately 2–7 cm long, with a hairy topside and subglobular fruits 0.5–1.2 cm in diameter (Figure 1A, Figure S1, and Table S1). These fruits ripen between June and September, offering a sweet, slightly tart flavor ideal for fresh consumption or processing into pies and jams [6]. *P. tomentosa* exhibits superior performance in cold tolerance, withstanding temperatures as low as −40°C [8], and is, therefore, a promising candidate for ornamental and fresh fruit production in cold regions. Despite this, the mechanisms governing its cold tolerance and the potential application of molecular markers remain largely unexplored.

**Figure 1:**
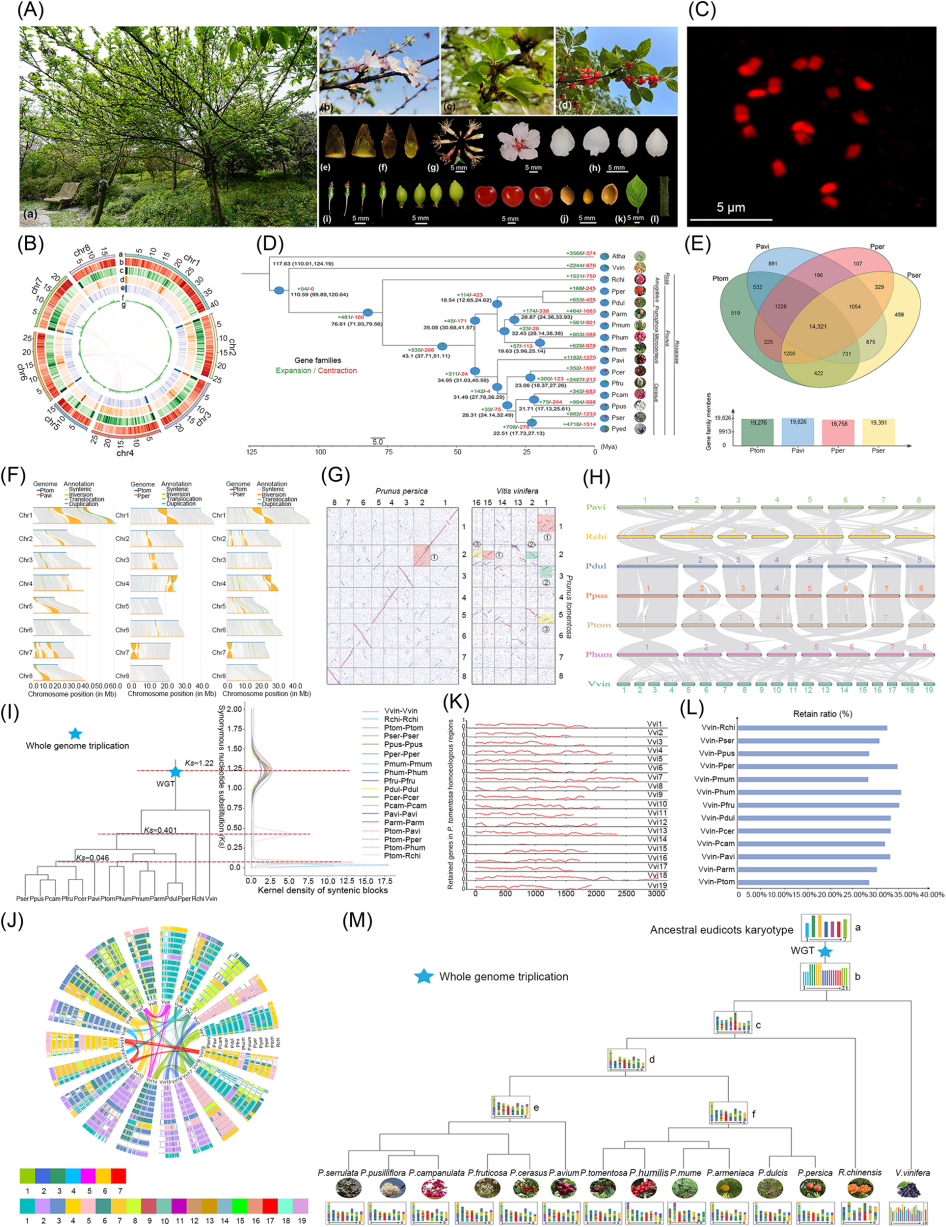
Phenotypic characterization, *de novo* genome assembly, and genomic analysis of *Prunus tomentosa*. (A) *P. tomentosa* phenotypic characterization. Various phenotypes of *P. tomentosa* were collected from January to July in 2021 and 2024, encompassing (a) individual panorama, (b) blooming flowers, (c) green and (d) ripe fruits, (e) leaf and (f) flower buds, (g) floral organ, (h) petal, (i) fruits at different stages of development, (j) seeds, (k) leaves, and (l) fruit stalks. (B) Summary of *P. tomentosa de novo* genome assembly and sequencing analysis. Moving from the outside to the inside, the tracks indicate (a) chromosome size (Mb), (b) gene and (c) repeat density (300 kb sliding window), (d) *Gypsy* density (300 kb sliding window), (e) *Copia* density (300 kb sliding window), (f) GC content (300 kb sliding window), and (g) synteny blocks among *P. tomentosa* chromosomes. (C) Karyotype of *P. tomentosa*. (D) Phylogenetic tree, divergence time, and profiles of contracted and expanded gene families in Ptom and 15 other plant species. (E) Venn diagram illustrating shared and unique gene families among four *Prunus* genomes. (F) Structural‐variant detection between *P. tomentosa*, and *P. avium*, *P. persica*, or *P. serrulata*. (G) Dotplots of homologous genes between *P. tomentosa* and *Vitis vinifera*, and between *P. tomentosa* and *P. persica*. (H) Syntenic diagram between *P. tomentosa* and other related species. Syntenic blocks are marked using gray lines. (I) Species phylogenetic trees for *Prunus* species. (J) Global alignment of homologous regions in *Prunus* plants with the grape as a reference. Collinear genes between each subgenome of species and grape are presented in each circle. The curved lines of the inner circle are composed of 19 grape chromosomes corresponding to the 7 chromosomes of the ancient core‐eudicot hexaploid ancestor (γ event). (K) Retention of duplicated genes residing in the *P. tomentosa* genome along with each grape chromosome. (L) Summary of gene retention in the grape genome compared with that of other *Prunus* species. (M) Chromosome karyotype reconstruction in *Prunus* species. The blue pentagram represents the whole‐genome triplication (WGT) event. The chromosome karyotype of the key nodes and number of chromosomes of each species are indicated in the diagram, and the name of each species is given, represented by its Latin name. Atha, *Arabidopsis thaliana*; Vvin, *V. vinifera*; Rchi, *Rosa chinensis*; Pper, *Prunus persica*; Pdul, *Prunus dulcis*; Parm, *Prunus armeniaca*; Pmum, *Prunus mume*; Phum, *Prunus humilis*; Ptom, *P. tomentosa*; Pavi, *P. avium*; Pcer, *Prunus cerasus*; Pfru, *Prunus fruticosa*; Pcam, *Prunus campanulata*; Ppus, *Prunus pusilliflora*; Pser, *Prunus serrulata*; Pyed, *Prunus yedoensis*; Chr 1–8, chromosomes 1–8.

We assembled a high‐quality chromosome‐scale *P. tomentosa* genome comprising 211.55 Mb, with 25,991 protein‐coding genes, 99.09% of which were functionally annotated. Repetitive sequences constituted 41.53% of the genome. *P. tomentosa* showed close genetic affinity to *P. humilis* (Phum), from which it diverged approximately 19.63 million years ago (Mya). Several syntenic regions, translocations, inversions, and duplications were revealed between *P. tomentosa* and other *Prunus* species, highlighting their genetic diversity and complexity. *P. tomentosa* underwent a whole genome triploidization event 115–130 Mya (*Ks* = 1.22), shared by core eudicots. Combined analysis of the transcriptome and metabolome identified inositol monophosphatase 3 and myoinositol oxygenase 1‐like as key factors in cold tolerance. These findings enhance our understanding of the complex *P. tomentosa* genetics, genome evolution, and adaptation to cold environments, and support further research in molecular breeding.

## RESULTS AND DISCUSSION

### Sequencing, assembly, and annotation of the *P. tomentosa* genome

We obtained 85.94 and 55.26 Gb of Illumina short‐read and Oxford Nanopore Technology (ONT) long‐read data, respectively (Table S2). Before the *de novo* assembly of *P. tomentosa*, the genome was estimated to be 232.19 Mb using a *k*‐mer survey (*k* = 23) from Illumina short reads, which closely matched the testing value (210.46 Mb) determined via flow cytometry (Figure S2 and Table S3). We assembled the genome at the chromosomal level with 109.94 Gb of Hi‐C reads (Table S2). This assembly resulted in 172 scaffolds covering 211.55 Mb, with scaffold N50 of 25.22 Mb and contig N50 of 3.40 Mb (Figure 1B and Table S4). Notably, its genome size was smaller than those of the *P. serrulata* (Pser) (265.4 Mb) [9], *P. campanulata* (Pcam) (280.2 Mb) [10], *P. conradinae* (Pcon) (289.62 Mb) [11], *P. pusilliflora* (Ppus) (309.62 Mb) [5], and *P. avium* (Pavi) (341.62 Mb) [12] genomes. Benchmarking Universal Single‐Copy Orthologs (BUSCO) analysis showed 98.5% completeness, with only 0.9% missing BUSCOs (Table S5). Moreover, we anchored 201.58 Mb, approximately 95.29% of the genome, to eight pseudochromosomes (Table S6). The Hi‐C heatmap revealed that its assembly is good, demonstrating well‐connected pseudochromosomes along the diagonal (Figure S3). The somatic cells of *P. tomentosa* contained 16 chromosomes as determined by the cytological observation method (Figure 1C). We identified 83.72 Mb of repetitive sequences (~39.58% of the genome), which included tandem repeats and transposable elements (TEs; Table S7). Notably, the most abundant long terminal repeat (LTR) retrotransposons were LTR/*Gypsy* and LTR/*Copia*, comprising 10.51% and 7.59% of total retrotransposons, respectively (Table S7). The repetitive sequence rate (~39.58%) in the *P. tomentosa* genome was lower than those in Pser (49.02%) [9], Pcam (50.33%) [10], Pcon (~46.23%) [11], Ppus (49.08%) [5], and Pavi (~59.29%) [12]. These findings may explain the relatively smaller genome size of *P. tomentosa* compared with those of other *Prunus* species. We identified 25,991 protein‐coding genes in the *P. tomentosa* genome. The BUSCO completeness metrics demonstrated high concordance, with the *P. tomentosa* genome displaying 98.5% completeness, and the annotated gene set indicating 97.7% completeness based on gene annotation (Table S8). Furthermore, 25,754 genes (~99.09%) were functionally annotated across various databases (Table S9). We demonstrated a LTR assembly index of 12.50 for the *P. tomentosa* genome (Table S10), indicating the high quality of the genome assembly.

### Gene family expansion/contraction analysis and divergence time estimation

We analyzed gene family dynamics between *P. tomentosa* and 15 other species, comparing unique paralogs, multiple‐ and single‐copy orthologs, other orthologs, and unclustered genes (Figure S4 and Table S11). In *P. tomentosa*, we observed 629 gene families expanding and 978 contracting during speciation from Phum (Figure 1D). *P. tomentosa* exhibited more expanded and contracted gene families than other *Prunus* species, such as *P. persica* (Pper), *P. mume* (Pmum), and *P. campanulata* (Pcam). Expanded, unique, and contracted family genes were significantly enriched (*p* < 0.05) in 2464, 174, and 2014 GO terms, respectively (Figures S5–S7 and Tables S12–S14). Expanded genes were notably enriched in *myo*‐inositol (MI) transport (GO:0015798) and MI transmembrane transporter activity (GO:0005365) (Figure S5 and Table S12). Using the TimeTree database (http://www.timetree.org/) and fossil calibration of known species, we estimated that *P. tomentosa* diverged from its common ancestor with Phum approximately 19.63 Mya (95% highest posterior density [HPD] of 3.96–25.14 Mya; Figure 1D). The divergence between two *Microcerasus* species (*P. tomentosa* and Phum) and two *Prunophora* species (Parm and Pmum) was estimated at about 32.43 Mya (95% HPD of 28.14–38.38 Mya).

### Evolution and structural variant detection between *P. tomentosa* and other *Prunus* species

We compared *P. tomentosa* to 15 other plant species to explore genome evolution using *Vitis vinifera* and *Arabidopsis thaliana* as outgroups. Phylogenetic analysis revealed that *P. tomentosa* was closely related to *P. humilis*, with both belonging to the subgenus *Microcerasus*. Furthermore, two species (*P. tomentosa* and Phum) of subgenus *Microcerasus* and two species (Pmum and Parm) of subgenus *Prunophora* were located on two branches and clustered with two species (Pper and Pdul) of subgenus *Amygdalus*, followed by seven species from the subgenus *Cerasus* (Figure 1D). Their genetic makeup aligned more closely with the subgenus *Prunophora* than with that of the subgenus *Cerasus*, which is consistent with findings from a previous genetic diversity analysis [13, 14, 15]. Consequently, it is proposed that *P. tomentosa* and Phum have been reclassified into the subgenus *Prunophora*, challenging earlier morphology‐based classifications [6, 16]. The four *Prunus* species shared 14,321 gene families, and *P. tomentosa* contained more unique gene families (519) than those of Pser (459) or Pper (107) (Figure 1E).

To compare the structural variations between *P. tomentosa* and multiple *Prunus* species, we identified syntenic regions, duplications, translocations, inversions, and unaligned genomic segments. Our findings revealed significant syntenic blocks between *P. tomentosa* and each of the compared species—140.71 Mb for Pavi, 129.14 Mb for Pper, and 123.49 Mb for Pser—indicating their evolutionary conservation (Tables S15–S17). Additionally, we analyzed genomic rearrangements, including inversions (21.66 Mb for Pavi, 18.69 Mb for Pper, and 16.28 Mb for Pser), translocations (4.65 Mb for Pavi, 2.60 Mb for Pper, and 8.56 Mb for Pser), and duplications (0.52 Mb for Pavi, 1.03 Mb for Pper, and 1.25 Mb for Pser) with each comparison (Figure 1F). A notable aspect of our analysis was the considerable portion of the unaligned genome in each comparison (37.49, 50.76, and 58.35 Mb), highlighting the genetic diversity and complexity among these species (Tables S15–S17). These findings contribute to our understanding of genomic architecture and evolutionary relationships within and between these species, highlighting the importance of genomic rearrangements in species evolution and adaptation. However, there exist a greater number of these genetic variations, such as inversions and translocations, between the genomes of *P. tomentosa* and Pavi compared with those between the *P. tomentosa* and Pper genomes (Figure 1F). One possible explanation is that Pavi may have experienced more intense artificial selection compared with that of Pper and Pser, leading to the observed chromosomal variations. However, further research is needed to substantiate these observations.

### Synteny analysis and polyploidization of *Prunus* plants

We explored polyploidization events in *Prunus* plants using homologous dot plots between species. Initially, a comparison between grapes and *P. tomentosa* revealed that three grape chromosomes corresponded to three *P. tomentosa* chromosomes, showing a 1:1 ratio of red fragments (Figure 1G). The results indicated that *P. tomentosa* underwent a single whole‐genome triplication event known as the gamma (γ) event, which is a common duplication event shared by core eudicots. *P. persica* experienced one whole‐genome triplication event according to the previous report [17]. Further syntenic analysis showed a 1:1 homologous ratio between *P. persica* and *P. tomentosa*, indicating that *P. tomentosa* likely underwent a comparable polyploidization event (Figure 1G). Extended homology analysis across multiple *Prunus* species (*P*. *tomentosa*, Pavi, *Prunus dulcis*, Ppus, and Phum) and two reference species (*Rosa chinensis* and *V. vinifera*) confirmed a consistent 1:1 homology ratio (Figure 1H). The synonymous nucleotide substitution rate was then calculated using data from 12 *Prunus* species and 2 reference plants, which revealed only 1 peak across these species, affirming that the *Prunus* lineage underwent just the γ event common to core eudicots (Figure 1I, Figures S8, S9, and Table S18). Variations in evolutionary rates across different species could potentially skew estimates of evolutionary time. To address this, shared evolutionary events were used as a benchmark for correction, aiming to deduce a more accurate timeline for species evolution. By employing the *Ks* value of the γ event in grapes as a reference, the *Ks* values for homologous genes were adjusted across all *Prunus* species, and the *Ks* peak was fitted (Table S19). This analysis showed the gamma event to have occurred between 115 and 130 Mya, with a *Ks* value of 1.22.

### Syntenic and gene retention analyses of *Prunus* plants

The intergenomic collinearity was assessed between various *Prunus* species and the grape genome, following the whole‐genome triplication in *Prunus* (Table S20). Analysis of collinear regions between grape and *Prunus* species revealed a 1:1 ratio, indicating the occurrence of only ancient gamma (γ) events. Using the grape genome as a reference, collinearity comparisons for each *Prunus* species were performed (Figure 1J). Furthermore, the gene retention within the homologous regions was analyzed by comparing the genomes of *Prunus* species with those of the grape (Table S21). The overall gene retention rate for the *Prunus* species was relatively high and ranged from 28% to 36%, likely associated with a whole‐genome triplication event. The genomic stability observed throughout the species was strongly linked to reduced rates of gene loss (Figure 1K,L).

### Ancestral chromosome reconstruction of *Prunus* plants

After undergoing polyploidization, fusion, and translocation events, the reconstruction of ancestral chromosomes provides significant insights into the evolutionary processes of species and the potential impacts of chromosome rearrangements. This analysis enhances our understanding of chromosome structure and function and clarifies the relationships among species. By comparing the genomes of 12 *Prunus* species, one Rosaceae species (*R. chinensis*), and one reference species (*V. vinifera*), we reconstructed the ancestral chromosomes from these collinearity genes. Through genome structure analysis of the *Prunus* species and comparison of dot plots between these species (Figures S10–S12), a clear 1:1 homologous relationship was evident. This observation enabled us to infer that the ancestral chromosomes of *Prunus* consisted of eight chromosomes (Figure 1M). Further analysis revealed that no chromosome fusion occurred during the transition from the ancestral state to the current chromosomes of *Prunus* species. Instead, translocations involving cross‐exchanges between chromosomes occurred. Consequently, there was no significant change in the number of chromosomes (Figure 1M).

### *PtIMP3* and *PtMIOX1L* maintain higher inositol contents in *P. tomentosa*, potentially conferring superior cold tolerance

**Figure 2:**
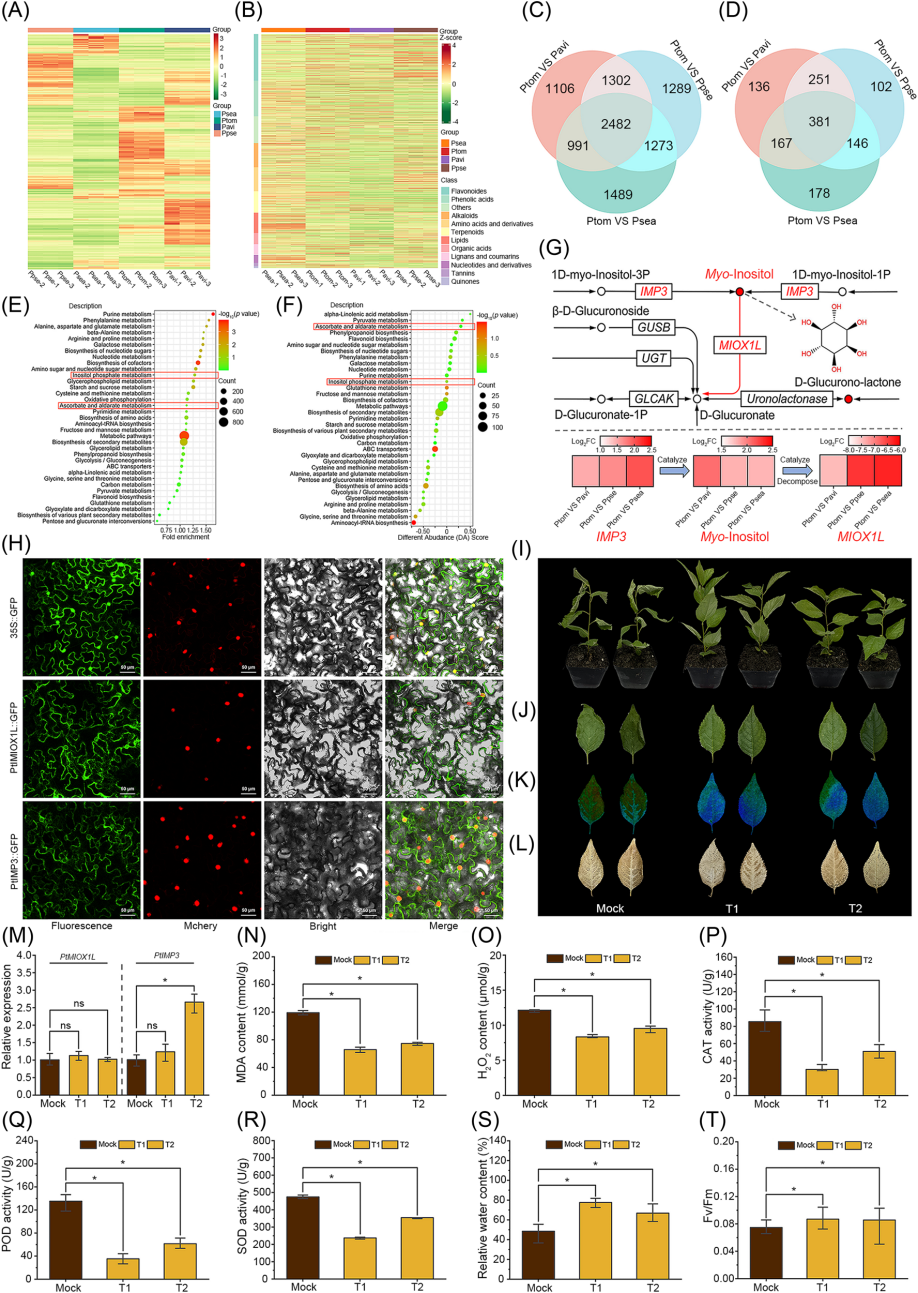
Combined analysis of transcriptome and metabolome, functional analysis of key differential metabolites, and subcellular localization of key differentially expressed genes. (A) Heat map displaying differential gene expression across Ppse, Psea, Ptom, and Pavi. (B) Heat map of differential metabolites (vertical for metabolites, classification by chemical compound). (C) Venn diagram showing significantly different genes among Ppse, Psea, Ptom, and Pavi. (D) Venn diagram illustrating significantly different metabolites in Ppse, Psea, Ptom, and Pavi. (E) KEGG enrichment analysis of differential genes. (F) KEGG enrichment analysis of differential metabolites. (G) Genes and metabolites related to ascorbate, aldarate, and inositol phosphate metabolism were enriched in both transcriptome and metabolome according to KEGG enrichment analysis. *Myo*‐inositol, *IMP3*, and *MIOX1L* were significantly expressed across all studied species (refer to KEGG pathways). (H) Subcellular localization of PtMIOX1L and PtIMP3. (I) After exogenous inositol treatment, phenotypic characteristics of cherry rootstocks at 2°C (acquisition time of 8 h after 2°C processing). (J) Leaf phenotype. (K) Chlorophyll fluorescence. (L) Histochemistry assay of H_2_O_2_ content (DAB staining). (M) Changes in the relative expression of *PtMIOX1L* and *PtIMP3* genes in cherry rootstocks were observed after spraying exogenous inositol. (N) MDA content. (O) H_2_O_2_ content. (P) CAT activity. (Q) POD activity. (R) SOD activity. (S) Relative water content in leaves. (T) Chlorophyll fluorescence parameters. Data for transcriptome and metabolome analyses (A–F) are presented for three replicates. In (M), data are presented as the mean ± standard deviation (SD) of three technique replicates. In (N–R), data are presented as the mean ± SD of five technique replicates. In (S) and (T), data are presented as the mean ± SD of five biological replicates. *p < 0.05, Student's *t*‐test.

Cold stress often exerts a significantly adverse impact on the growth and development of plants, resulting in decreased total chlorophyll synthesis and elevated levels of reactive oxygen species (ROS), leading to cellular membrane lipid peroxidation and an excessive production of malondialdehyde (MDA) [18]. The superior performance of *P. tomentosa* in tolerating low temperatures necessitates further analysis of its specific transcriptional and metabolic regulatory networks in response to cold stress. In this study, we generated transcriptional and metabolic profiles of four *Prunus* species: *P. pseudocerasus* (Ppse), *P. serrula* (Psea), *P. tomentosa*, and Pavi, identifying 2482 common significant differentially expressed genes (DEGs) primarily related to metabolic pathways, biosynthesis of secondary metabolites, and biosynthesis of cofactors, and 381 common significantly differential metabolites associated with metabolic pathways, secondary metabolite biosynthesis, and ABC transporters (Figure 2A–D and Tables S22–S27). The KEGG enrichment analysis of these differentials revealed that the molecular functions of differential genes and metabolites co‐enriched in the inositol phosphate, ascorbate, and aldarate metabolism pathways (Figure 2E,F). PtIMP3 catalyzes the conversion of 1D‐*myo*‐inositol‐1P/1D‐*myo*‐inositol‐3P to *myo*‐inositol and displays significantly higher expression levels in *P. tomentosa* compared to three cold‐sensitive *Cerasus* species, namely Pavi, Ppse, and Psea (Figure 2G). Additionally, PtMIOX1L catalyzes *myo*‐inositol conversion to d‐glucuronate, showing lower expression levels than those of other three *Prunus* species (Figure 2G). Analysis of differential metabolites indicated that *myo*‐inositol was notably more abundant in *P. tomentosa* compare to other three *Prunus* species (Figure 2G). Specifically, the high expression abundance of *PtIMP3* and low expression abundance of *PtMIOX1L* resulted in higher endogenous inositol levels in *P. tomentosa* compared with that in the three cold‐sensitive *Cerasus* species (Figure 2G). This finding supports the notion of *P. tomentosa*'s adaptation to extreme environmental conditions, contributing to a better comprehension of the evolutionary and functional roles of these genes. However, the lack of supporting molecular evidence emphasizes the necessity for additional research to validate and extend these discoveries.

The subcellular localization showed that PtIMP3 and PtMIOX1L were expressed in the cytoplasm, consistent with the predictions obtained by CELLO v.2.5 (Figure 2H and Table S28). In addition, we investigated the physiological function of differentially expressed metabolite *myo*‐inositol against cold stress in cherries (Figure 2I–T). Phenotypic observation showed that the exogenous application with different concentrations of *myo*‐inositol enhanced the cold tolerance of cherry rootstocks (Figure 2I–L). Exogenous application of *myo*‐inositol led to a significant reduction in MDA and hydrogen peroxide (H_2_O_2_) contents, and catalase (CAT), peroxidase (POD), and superoxide dismutase (SOD) activities (Figure 2N–R). Relative water content (RWC) increased significantly in both treatment groups of *myo*‐inositol (Figure 2S). Finally, chlorophyll fluorescence (Fv/Fm), a marker of photosynthetic efficiency, significantly improved in both treatment groups (Figure 2T). These results suggest that *myo*‐inositol may function as a compatible solute, protection against cold stress by regulating concentrations of ROS and maintaining stable RWC. These findings indicate its effective role in regulating ROS concentrations and maintaining cell turgor by binding water molecules, aligning with earlier report [19].

## METHODS

Comprehensive detailed procedures are provided in the Supplementary Information.

## AUTHOR CONTRIBUTIONS

**Songtao Jiu**: Project administration; conceptualization; investigation; formal analysis; writing—review and editing. **Muhammad Aamir Manzoor**: Investigation; formal analysis; writing—review and editing. **Zhengxin Lv**: Investigation; formal analysis. **Baozheng Chen**: Investigation; methodology; formal analysis. **Shaoqin Shen**: Investigation; formal analysis. **Yan Xu**: Validation; visualization. **Moyang Liu**: Project administration; Methodology. **Chengwei Li**: Investigation. **Xunju Liu**: Investigation. **Yanhong Fu**: Formal analysis. **Qijing Zhang**: Resources. **Ruie Liu**: Resources. **Xinyu Zhang**: Visualization. **Shiping Wang**: Project administration. **Xiaoming Song**: Methodology; Conceptualization; project administration; supervision. **Yang Dong**: Methodology; project administration; supervision. **Caixi Zhang**: Project administration; funding acquisition; conceptualization; resources; supervision.

## CONFLICT OF INTEREST STATEMENT

The authors declare no conflicts of interest.

## ETHICS STATEMENT

No animals or humans were involved in this study.

## Supporting information


**Figure S1.** *P. tomentosa* fruit parameters, including (A) single fruit weight, (B) hardness, (C) titratable acid, (D) total soluble solids, and (E) longitudinal and transverse diameters of fruits.
**Figure S2.** Estimation of *Prunus tomentosa* genome size.
**Figure S3.** High‐resolution Hi‐C contact matrix in the chromosome‐level assembly of the *Prunus tomentosa* genome.
**Figure S4.** Gene number distribution of single‐ and multiple copies and other orthologs, including unique paralogs and unclustered genes in *Arabidopsis thaliana* (Atha), *Vitis vinifera* (Vvin), *Rosa chinensis* (Rchi), *Prunus persica* (Pper), *Prunus dulcis* (Pdul), *Prunus armeniaca* (Parm), *Prunus mume* (Pmum), *Prunus humilis* (Phum), *Prunus avium* (Pavi), *Prunus cerasus* (Pcer), *Prunus fruticosa* (Pfru), *Prunus campanulata* (Pcam), *Prunus pusilliflora* (Ppus), *Prunus serrulata* (Pser), *Prunus yedoensis* (Pyed), and *P. tomentosa* (Ptom).
**Figure S5.** GO enrichment analysis for the expanded gene families in *Prunus tomentosa*.
**Figure S6.** GO enrichment analysis for the contracted gene families in *Prunus tomentosa*.
**Figure S7.** GO enrichment analysis for the unique gene families in *Prunus tomentosa*.
**Figure S8.** *Ks* density curve before correction.
**Figure S9.** Distribution map of *Ks* density between species.
**Figure S10.** Homologous dotplots between *Prunus avium* and *Prunus tomentosa*.
**Figure S11.** Homologous dotplots between *Rosa chinensis* and *Prunus tomentosa*.
**Figure S12.** Homologous dotplots between *Prunus tomentosa* and ancestors of core eudicots.

**Table S1.** Phenotypic characteristics of flower and fruit organs in *Prunus tomentosa*.
**Table S2.** Data statistics of whole‐genome sequencing for *Prunus tomentosa*.
**Table S3.** The estimation of genome size of *Prunus tomentosa* using flow cytometry.
**Table S4.** Statistics for the *Prunus tomentosa* assembly.
**Table S5.** Completeness of the genome assembly measured by Benchmarking Universal Single‐Copy Orthologs (BUSCO).
**Table S6.** Data statistics of ordering and orienting the scaffolds on 8 pseudomolecules.
**Table S7.** Statistics of repetitive sequence classification from the *Prunus tomentosa* genome.
**Table S8.** Completeness of the assembly and the annotated genes measured by Benchmarking Universal Single‐Copy Orthologs (BUSCO).
**Table S9.** Statistics of gene functional annotation in the *Prunus tomentosa* genome.
**Table S10.** Long‐terminal‐repeat assembly index (LAI) analysis and contig N50s of different genome assemblies in *Prunus* species.
**Table S11.** Comparison of gene families' statistics between *Prunus tomentosa* and other species.
**Table S12.** Gene ontology (GO) enrichment analysis of the expanded gene families in *Prunus tomentosa*.
**Table S13.** Gene ontology (GO) enrichment analysis of the contracted gene families in *Prunus tomentosa*.
**Table S14.** Gene ontology (GO) enrichment analysis of the unique gene families in *Prunus tomentosa*.
**Table S15.** Structural‐variant detection between *Prunus tomentosa* and *P. avium* genomes.
**Table S16.** Structural‐variant detection between *Prunus tomentosa* and *P. persica* genomes.
**Table S17.** Structural‐variant detection between *Prunus tomentosa* and *P. serrulata* genomes.
**Table S18.** Kernel function analysis of *Ks* distribution related to duplication events within each genome and between genomes (before evolutionary rate correction).
**Table S19.** Kernel function analysis of *Ks* distribution related to duplication events within each genome and between genomes (after evolutionary rate correction).
**Table S20.** The collinear list of 12 species.
**Table S21.** The percentage of retained genes in the homologous regions of each species compared with *Vitis vinifera*.
**Tables S22.** The significant differential expression genes in Psea vs. Ptom.
**Tables S23.** The significant differential expression genes in Pavi vs. Ptom.
**Tables S24.** The significant differential expression genes in Ppse vs. Ptom.
**Table S25.** KEGG enrichment analysis of differential metabolites in Psea vs. Ptom
**Table S26.** KEGG enrichment analysis of differential metabolites in Pavi vs. Ptom.
**Table S27.** KEGG enrichment analysis of differential metabolites in Ppse vs. Ptom.
**Table S28.** Primer sequences used in this study.

## Data Availability

The data that supports the findings of this study are available in the supplementary material of this article. The raw genome sequencing data for *P. tomentosa* are available at the National Genomics Data Center (https://ngdc.cncb.ac.cn/gsa/browse/CRA015735). The data of transcriptome and metabolome sequencing were uploaded to the NCBI Sequence Read Archive (SRA) database with the accession number PRJNA1230348 (https://www.ncbi.nlm.nih.gov/bioproject/?term=PRJNA1230348). The data and scripts used are saved in GitHub https://github.com/ericchenbz/The-genome-of-the-Prunus-tomentosa. Supplementary materials (methods, figures, tables, graphical abstract, slides, videos, Chinese translated version, and update materials) may be found in the online DOI or iMeta Science http://www.imeta.science/.
