# Supplementary material for: New insights into genome assembly at the chromosome‐level of Prunus tomentosa in evolution and cold tolerance
Source: Imeta. 2025 Mar 20;4(2):e70016. doi: 10.1002/imt2.70016 (PMC11995182; doi:10.1002/imt2.70016)
Supplement: Supplementary file 1 — Figure S1. P. tomentosa fruit parameters, including (A) single fruit weight, (B) hardness, (C) titratable acid, (D) total soluble solids, and (E) longitudinal and transverse diameters of fruits. Figure S2. Estimation of Prunus tomentosa genome size. Figure S3. High‐resolution Hi‐C contact matrix in the chromosome‐level assembly of the Prunus tomentosa genome. Figure S4. Gene number distribution of single‐ and multiple copies and other orthologs, including unique paralogs and unclustered genes in Arabidopsis thaliana (Atha), Vitis vinifera (Vvin), Rosa chinensis (Rchi), Prunus persica (Pper), Prunus dulcis (Pdul), Prunus armeniaca (Parm), Prunus mume (Pmum), Prunus humilis (Phum), Prunus avium (Pavi), Prunus cerasus (Pcer), Prunus fruticosa (Pfru), Prunus campanulata (Pcam), Prunus pusilliflora (Ppus), Prunus serrulata (Pser), Prunus yedoensis (Pyed), and P. tomentosa (Ptom). Figure S5. GO enrichment analysis for the expanded gene families in Prunus tomentosa. Figure S6. GO enrichment analysis for the contracted gene families in Prunus tomentosa. Figure S7. GO enrichment analysis for the unique gene families in Prunus tomentosa. Figure S8. Ks density curve before correction. Figure S9. Distribution map of Ks density between species. Figure S10. Homologous dotplots between Prunus avium and Prunus tomentosa. Figure S11. Homologous dotplots between Rosa chinensis and Prunus tomentosa. Figure S12. Homologous dotplots between Prunus tomentosa and ancestors of core eudicots. [file IMT2-4-e70016-s001.docx]

Supporting information to

**New insights into genome assembly at the chromosome-level of** ***Prunus tomentosa* in evolution and cold tolerance**

**Running title:** Genomic insights into evolution and cold tolerance of *Prunus tomentosa*

Songtao Jiu^1, 6^**^#^**^*^, Muhammad Aamir Manzoor^1^**^#^**, Zhengxin Lv^1^**^#^**, Baozheng Chen^2^**^#^**, Shaoqin Shen^3^**^#^**, Yan Xu^1^, Moyang Liu^1^, Chengwei Li^4^, Xunju Liu^1^, Yanhong Fu^3^, Qijing Zhang^5^, Ruie Liu^1^, Xinyu Zhang^1^, Shiping Wang^1^, Xiaoming Song^3*^, Yang Dong^2*^, Caixi Zhang^1*^

^1^ Department of Plant Science, School of Agriculture and Biology, Shanghai Jiao Tong University, Shanghai 200240, P. R. China

^2^ Province Key Laboratory, Biological Big Data College, Yunnan Agricultural University, Kunming 650201, P. R. China

^3^ College of Life Sciences, North China University of Science and Technology, Tangshan 063210, P. R. China

^4^ Department of Molecular, Cellular & Developmental Biology, College of Arts and Sciences, University of Colorado Boulder, Colorado 80309, United States

^5^ Liaoning Institute of Pomology, Liaoning Academy of Agricultural Sciences, Yingkou 115009, P. R. China

**^#^** Songtao Jiu, Muhammad Aamir Manzoor, Zhengxin Lv, Baozheng Chen, and Shaoqin Shen contributed equally to this study.

***Correspondence:** [acaizh@sjtu.edu.cn](mailto:acaizh@sjtu.edu.cn) (Caixi Zhang); [jiusongtao@sjtu.edu.cn](mailto:jiusongtao@sjtu.edu.cn) (Songtao Jiu); [songxm@ncst.edu.cn](mailto:songxm@ncst.edu.cn) (Xiaoming Song); [loyalyang@163.com](mailto:loyalyang@163.com) (Yang Dong)

**Supplementary materials and methods**

**Plant materials and treatment**

To facilitate the *P. tomentosa* genomic sequencing and assembly, fresh leaf and ripe fruit samples were collected from a mature tree in the Shanghai Botanical Garden, Shanghai, China (121°27'4" N, 31°9'14" E). Single fruit weight, fresh seed weight, longitudinal and transverse fruit diameters, and total soluble solids of ripe fruit from *P. tomentosa* were determined. The hardness of *P. tomentosa* fruits was evaluated using a Texture Analyzer (TXT Plus, Stable Micro Systems, Surrey, UK) with the following settings: P/50 flat probe, 5 mm/s pre-test speed, 5 mm/s post-test speed, 5 s pause time between cycles, 5 g trigger force, 1.0 mm/s test speed, and 2.5 mm test distance. The titratable acid of the ripe fruits was measured using the method described by Kazemi et al. (2011) [1] and expressed as a percentage of anhydrous malic acid. The ripe fruit samples of *P. avium* and *P. pseudocerasus* were collected at the orchard of Shanghai Jiao Tong University, Shanghai, China (121°26ʹ54ʺ N, 31°2ʹ19ʺ E). The ripe fruit samples from a wild *P. serrula* tree were gathered in its natural habitat, located in Shangri-La City, Diqing Tibetan Autonomous Prefecture, Yunnan Province, China (99°49ʹ7ʺ N, 27°49ʹ 28ʺ E). The 2-month-old cherry rootstocks *cv*. ‘Gisela 6’ were cultivated in pots at 26 °C under a 16:8 h light:dark (L:D) cycle at Shanghai Jiao Tong University, Shanghai, China. Before cold treatment, all seedlings were regularly irrigated and subjected to three treatments randomly: sprayed with water (control; Mock), 400 ppm *myo*-inositol (T1), and 800 ppm *myo*-inositol (T2). The leaves were sprayed until water droplets dripped from them, with spraying performed four times every 6 h. To assess the cold resistance of the different treatment groups, the seedlings were initially placed in low-temperature incubators, with temperatures gradually lowered to 20 °C for 12 h, 16 °C for 6 h, and finally 12 °C for 6 h to acclimatize them to low temperatures. The seedlings were subjected to cold stress at 2 °C for 8 h. Following this, leaf samples from the three groups were collected after the cold treatment, frozen in liquid nitrogen, and stored at −80 °C. Each treatment group comprised eight seedlings. Five leaves were collected from the middle section of each seedling for phenotypic assessment and subsequent analysis.

**Karyotype analysis of *P. tomentosa.***

To determine the karyotype of *P. tomentosa*, we followed the procedures outlined by Wang et al. (2024) [2]. Initially, healthy seeds underwent water selection and disinfection treatment. Subsequently, they were soaked in a culture box at 25 °C. Upon reaching root growth of 0.5‒1.0 cm, root tips were harvested at around 9:30 am. The collected root tips were pretreated with 0.002 M 8-hydroxyquinoline in the dark at 15 °C for 4 h, followed by fixation in a solution of 3:1 absolute alcohol:glacial acetic acid. After fixation, the root tips were hydrolyzed in 1 M HCl at 60 °C for 5 min, then trimmed to approximately 1 mm using a blade. They were subsequently stained with Schiff’s reagent for 20 min. Finally, the stained samples were examined under a microscope to determine the chromosome numbers.

**DNA/RNA extraction, library preparation, and transcriptome sequencing**

The DNeasy Plant Kit (Tiangen Biotech Co. Ltd., Beijing, China) was used to extract high-quality genomic DNA. Total RNA was extracted using the same company’s plant RNA purification kit and treated with RNase-free DNase I (Takara, Bio Inc., Tokyo, Japan) to eliminate genomic DNA contamination. The purity and concentration of the DNA and RNA were determined using a Nanodrop 2000 spectrometer (Thermo Fisher Scientific, MA, USA) and Qubit 3.0 fluorometer (Thermo Fisher Scientific). Furthermore, the integrity of the DNA and RNA was assessed using 0.8% agarose gel electrophoresis using pulsed-field techniques. For transcriptome sequencing, poly (A) + RNA was separated from total RNA using Dynal oligo (dT) 25 beads (Thermo Fisher Scientific) according to the manufacturer’s protocol. The cDNA libraries of ripe fruit samples from the four species (*P. tomentosa*, *P. avium*, *P. pseudocerasus*, and *P. serrula*) were constructed as described by Quan et al. (2017) [3] and sequenced on a NovaSeq platform (Illumina, San Diego, CA, USA) by Wuhan Benagen Technology Co., Ltd., Wuhan, China.

**Genomic DNA sequencing**

*P. tomentosa* genome assembly was conducted using both long- and short-read sequencing data. A paired-end library for short-read sequencing was created using GenElute Plant Genomic DNA Miniprep kits (Sigma-Aldrich, Corp., MO, USA) and subsequently sequenced on an Illumina HiSeq X Ten platform (Illumina). The ONT library was constructed with the SQK-LSK109 ligation sequencing kit (Oxford Nano Technologies) following the manufacturer’s instructions. Long DNA fragments were selected using a BluePippin system (Sage Science, Beverly, MA, USA). The fragment ends were then repaired and ligated using the NEBNex Ultra II End Repair/dA-Tailing Module Kit (New England Biolabs, MA, USA). The sizes of the library fragments were quantified using a Qubit 3.0 Fluorometer (Thermo Fisher Scientific). ONT sequencing was performed using a PromethION 48 platform (Oxford Nanopore Technology) at Novogene Co., Ltd., Beijing, China. An Hi-C sequencing library was constructed following by the previous study [4], and sequenced on the Illumina HiSeq X Ten platform (Illumina).

**Genome assembly and evaluation**

The *P. tomentosa* genomic size was determined using flow cytometry with a BD FACSCalibur system (BD Biosciences, Franklin Lakes, NJ, USA), using *Oryza sativa* as an internal standard. Quality control of next-generation sequencing (NGS) data and whole-genome sequencing of paired-end reads were executed using Fastp v0.20.2 [5], with default settings for generating clean reads. For the Nanopore data, clean reads were assembled *de novo* using NECAT v0.0.1 [6], followed by three rounds of polishing with Racon [7]. All clean Illumina paired-end reads underwent two polishing iterations using Pilon v1.21 [8] with default settings. Redundant sequences were removed using purge_dup v1.2.5 to conduct contig assembly. Hi-C data was used to resolve assembly discrepancies and refine the scaffolding process to perform chromosomal sequences. Chromosomal-level assembly was conducted using HiC-Pro [9] and 3D-DNA v180922 [10], with orientations manually verified using Juicer v1.6.2 [11] based on Hi-C heatmaps. NGS data alignment to the genome assembly was performed using the Burrows-Wheeler Aligner with default parameters for coverage ratio statistics [12]. Genome integrity was assessed using LAI. The completeness of the assembly was evaluated using BUSCO v5.3.1 with default settings [13].

**Annotation of repetitive sequences**

Repetitive sequences in the genome were identified using *ab initio* and homology-based techniques. The *ab initio* approach involved LTR elements using LTR_FINDER v1.07 [14], LTRharvest v1.5.10 [15], and LTR_retriever v1.8.0 [16], all with default settings. Novel repetitive elements were identified using RepeatModeler v2.0.10 [17]. The repeat library was downloaded from Repbase v21.12 (<https://www.girinst.org/downloads/>) [18]. Further prediction of repetitive elements was performed using RepeatMasker v4.0.7 [19], utilizing both the Repbase database and *de novo* repeat libraries. Tandem repeats were annotated using Tandem Repeat Finder v4.09 [20].

**Gene prediction and functional annotation**

Protein-coding genes within the *P. tomentosa* genome were predicted using *ab initio*, homology-based, and transcriptome-based methods. *Ab initio* gene prediction tools included Augustus v3.0.3 [21], SNAP v2006-07-28 [22], GenScan v1.0 [23], and GlimmHMM v3.0.1 [24]. Homology-based gene prediction involved the utilization of *P. avium* proteins obtained from NCBI, which were analyzed using Exonerate v2.2.0 [25]. Transcriptome-based gene models were predicted based on RNA-seq sequences from related species, such as *P. avium (*PRJNA550274, PRJNA419491, PRJNA595502, and PRJNA73727), *Prunus cerasus* (PRJNA295439 and PRJNA327561), *Prunus pseudocerasus* (PRJNA260424), and *Prunus subhirtella* (PRJNA596558) using StringTie v1.3.4 [26] and the Program to Assemble Spliced Alignments [27]. These predictions were integrated using EvidenceModeler v1.1.1 [28].

Functional annotation of genes was based on sequence similarity and domain conservation, involving searches in databases, such as NR, KEGG, and Swiss-Prot using the BLAST tool, domain searches against Pfam via HMMER v3.0 [29], and GO term annotations using InterProScan 5 (EMBL-EBI, Cambridge, UK) [30]. Non-coding RNAs were predicted using tRNAscan-SE v1.3.1 for tRNA [31], RNAmmer v1.2 for rRNAs [32], and INFERNAL v1.1.2 for miRNAs and snRNAs [33], with additional ncRNAs predicted using Rfam v1.0.4 [34].

**Evolutionary and structural-variant analysis**

Orthologous genes were identified by comparing the complete genome sequences of *A. thaliana* (Atha) [35], *V. vinifera* (Vvin) [36], *R. chinensis* (Rchi) [37], *Prunus persica* (Pper) [38], *P. dulcis* (Pdul) [39], *P. armeniaca* (Parm) [40], *P. mume* (Pmum) [41], *P. huminis* (Phum; <https://ngdc.cncb.ac.cn/search/?dbId=gwh&q=GWHBCKI00000000>), *P. avium* (Pavi) [42], *P. cerasus* (Pcer) [43], Pfru [44], Pcam [45], *P. pusilliflora* (Ppus) [46], *P. serrulata* (Pser) [47], and *P. yedoensis* (Pyed) [48], with *P. tomentosa* (Ptom). Gene families were identified using OrthoFinder v2.2.7 with default settings. Single-copy orthologous gene alignments were performed using MUSCLE v5.1 [49] under default conditions. A maximum-likelihood phylogenetic tree was generated using PhyML v3.0 (Laboratoire d'Informatique, Montpellier, France) with default settings. Divergence times were estimated using the MCMCtree program within the PAML v4.9j package [50] and calibrated against established divergence times from TIMETREE (<http://www.timetree.org>) and fossil calibrations of known species, including *Arabidopsis* and *V. vinifera*. Furthermore, expansions and contractions of gene families were determined using CAFÉ v3.1 [51]. Structural variants were detected between *P. tomentosa* (Ptom) and three other *Prunus* genomes (Pavi, Pper, and Pser) using MUMmer v3.23 in conjunction with SyRI [52].

**Genome collinearity and visualization**

Genome collinearity was assessed using the whole-genome duplication integrated analysis (WGDI) [53]. First, BLASTP was used to identify homologous genes within and between the genomes of two species. The “-d” subroutine of WGDI was used to create homologous gene dotplots, and the “-icl” subroutine was used to detect syntenic genes with a maximal gap length of collinearity set at 50. Using the grape genome as a reference, collinear alignments for each species and lists of collinearities were constructed. Theoretically, owing to a WGT event [36], each grape gene has two additional collinear genes. In these alignments, cells contain a gene name if a collinear gene is detected and are marked with a dot if absent. The corresponding number of columns was assigned according to the WGD or WGT events for each species. Collinear alignments were visualized using a Circos plot created with the “-ci” module in WGDI [53]. Synteny and microsynteny among different species were visualized using MCscanX in Python [54].

***Ks* calculation and distribution fitting**

MUSCLE was used for homologous amino acid sequence comparison (maxiters 1-diags-sv-distance1 kbit20-3) [49]. The PAL2NAL program was used to convert protein comparisons to codon comparisons based on coding sequences (CDS) [55]. Subsequently, *Ka* and *Ks* were calculated using the yn00 program of PAML and the Nei-Gojobori method [56]. The median *Ks* between homologous genes in collinear blocks was used to categorize blocks resulting from duplication events. *Ks* values between co-linked gene pairs are displayed in a dot plot using the “-bk” parameter in WGDI, with different *Ks* value intervals represented by different colors. The distribution of *Ks* density was determined using the modules Kspeak, PeaksFit, and KsFigure. *Ks* density distribution curves were plotted using Kspeak. Multipeak fitting was then performed using PeaksFit (Systat Software, Inc., Chicago, IL, USA), and multiple fitted density curves were converted into a single plot using KsFigures.

***Prunus* karyotype inference**

After a species undergoes polyploidization, chromosome fusion and translocation commonly occur. The collinearity between genomes reflects changes in the karyotype and can infer the trajectory of chromosome evolution. We analyzed the genomes of *Prunus* plants and *R. chinensis* using homologous gene point maps to infer the ancestral *Prunus* chromosome karyotype. Previous studies suggest that current chromosomes at relatively new nodes are derived from the fusion of ancestral chromosomes through end-to-end joining of two different chromosomes or nested chromosome fusion, where one chromosome is inserted into another [42,57,58]. These changes reduce the number of chromosomes and result in gene loss.

**Transcriptomics analysis**

We obtained RNA-seq data from the ripe fruits of *P. tomentosa*, *P. avium*, *P. pseudocerasus*, and *P. serrula*, as previously reported [59]. Quality control of the RNA-seq reads was performed using FastQC v0.11.9 with default settings. Subsequently, the RNA-seq reads were mapped to the genome sequence using HISAT2 v2.1.0 with default settings [60]. DEGs were identified using DESeq2 v1.38.3 [61], applying a significance threshold of *p* < 0.05 and |log_2_ fold-change| > 1. GO analysis of DEGs was performed using the GOSeq R package v3.15 (RStudio, MA, USA), considering GO terms with a corrected *p* value < 0.05 as significantly enriched. Functional classification of the GO annotations was visualized using the web gene ontology annotation plot (WEGO Bioinformatics, Weihai, China). DEGs were aligned with KEGG orthologs in the KEGG pathway database (<http://www.kegg.jp/kegg/kegg1.html>) using blastall.

**Metabolomics analysis**

Metabolites were extracted from the ripe fruit samples of four cherry species and analyzed using ultra-performance liquid chromatography–tandem mass spectrometry (LC–MS) technology [62]. Chromatographic separation was performed on a BEH C18 column (Thermo Fisher Scientific), with detection in both positive and negative modes. Metabolites were identified via comparison with the Metlin and human metabolome databases, and differential metabolites were identified using orthogonal partial least squares discriminant analysis (Variable Importance in Projection > 1, *p* < 0.05).

**qRT-PCR**

The expression levels of *IMP3* and *MIOX1L* were determined using quantitative reverse transcriptase PCR (qRT–PCR) with three replicates according to our previously reported methods [63,64]. Then, cDNA was detected quantitatively on a BIOER LineGene 9600 Plus Fluorescent Quantitative Detection System (FQD-96A, BIOER, Hangzhou, China) using a TB Green^TM^ Premix Ex Taq^TM^ II kit (TaKaRa, Tokyo, Japan). Relative expression levels of genes were determined using the 2^-ΔΔT^ method, as previously described by Livak & Schmittgen (2001) [65], with *Actin* serving as the reference gene for normalization. The primer sequences of examined genes are listed in Table S28.

**Subcellular localization**

The cellular localization of PtoMIOX1L and PtoIMP3 were predicted using CELLO v2.5 (http://cello.life.nctu.edu.tw/). The CDS of *PtoMIOX1L* and *PtoIMP3*, excluding the stop codon, were amplified and cloned into the polyhydroxybutyrate (pHB) vector. This vector contained two cauliflower mosaic virus 35S promoters, a GFP fluorescent protein tag (GFP), and a translation enhancer. The resulting fusion constructs, p35S-*PtoMIOX1L*-GFP and p35S-*PtoIMP3*-GFP, were used for transformation as previously described [66,67]. Once fluorescence reached optimal levels, GFP fluorescence was observed 3–5 days post-infiltration using a Zeiss LSM 780 confocal laser scanning microscope (Carl-Zeiss, Oberkochen, Germany). The primer sequences for subcellular localization are listed in Table S28.

**Relative water content measurement**

Relative water content (RWC) was determined using the fresh weight method. Fresh leaves were cleaned, dried, and weighed to obtain the fresh weight (FW). They were then soaked in distilled water for 8 h to ascertain the turgid weight (TW). Next, the dry weight (DW) was measured after incubating the leaves at 60 ℃ for 24 h. RWC (%) was calculated using the following formula: RWC (%) = (FW–DW)/(TW–DW) × 100.

**Chlorophyll fluorescence measurement**

Chlorophyll fluorescence, expressed as Fv/Fm, was measured using an FMS-2 pulse-modulated fluorometer (Hansatech, London, UK). Samples were dark-adapted for 30 min before measuring the initial fluorescence (F0). Maximum fluorescence (Fm) was determined following a 0.7-s exposure to 5000 μmol m^−2^ s^−1^ pulsed light. Fv/Fm was calculated as Fv/Fm = (Fm–F0)/Fm.

**Measurement of antioxidant enzyme activities**

Leaf samples (25 mg) were homogenized in 5 mL of 50 mM sodium phosphate buffer (pH 7.8 [Thermo Fisher Scientific]) and centrifuged at 15,000 × *g* for 20 min at 4 ℃. The resulting enzyme extract was stored at 4 ℃. Superoxide dismutase (SOD), peroxidase (POD), and catalase (CAT) activities were measured using commercial kits (Solarbio Life Sciences, Beijing, China) according to the manufacturer’s protocol, with absorbance readings at 560, 470, and 240 nm, respectively.

**Measurement of MDA and H_2_O_2_ contents**

The malondialdehyde (MDA) content was determined using commercial kits (Sangon Biotech, Shanghai, China) following the manufacturer’s instructions, measuring absorbance at 532 and 600 nm. The hydrogen peroxide (H_2_O_2_) content was assessed with commercial kits (Sangon Biotech, Shanghai, China) according to the manufacturer’s protocol, with absorbance readings at 415 nm.

**Quantification and statistical analysis**

Data were analyzed using SAS v9.3 (SAS Institute Inc., Cary, NC, USA). Statistical significance was assessed using a two-tailed Student’s *t*-test. Results for fruit texture parameters are presented as the mean ± standard deviation (SD) based on at least eight replicates. RT-qPCR data are reported as the mean ± SD of three technical replicates. Enzyme activity assay data are expressed as the mean ± SD of five technical replicates. RWC and Fv/Fm data are reported as the mean ± SD of five biological replicates. Statistical significance was determined at *p* < 0.05.

**References**

1. Kazemi, Mohsen, Mehdi Aran, and Serveh Zamani. 2011. “Effect of salicylic acid treatments on quality characteristics of apple fruits during storage.” *American Journal of Plant Physiology* 6(2): 113–119. <https://doi.org/10.3923/ajpp.2011.113.119>
2. Wang, Nan, Chih-Chieh Yu, Yanxia Jia, and Yaowu Xing. 2024. “Ecological factors correlate with genome size variation of *Acanthocalyx* (Caprifoliaceae) in the Hengduan-Himalaya Mountains.” *Taiwania* 69(2): 142–150. <https://doi.org/10.6165/tai.2024.69.142>
3. Quan, Jine, Seng Meng, Erhui Guo, Sheng Zhang, Zhong Zhao, and Xitian Yang. 2017. “*De novo* sequencing and comparative transcriptome analysis of adventitious root development induced by exogenous indole-3-butyric acid in cuttings of tetraploid black locust.” *BMC Genomics* 18: 179. <https://doi.org/10.1186/s12864-017-3554-4>
4. Belton, Jon-Matthew, Rachel Patton McCord, Johan Harmen Gibcus, Natalia Naumova, Ye Zhan, and Job Dekker. 2012. “Hi-C: A comprehensive technique to capture the conformation of genomes.” *Methods* 58(3): 268–276. <https://doi.org/10.1016/j.ymeth.2012.05.001>
5. Chen, Shifu, Yanqing Zhou, Yaru Chen, and Jia Gu. 2018. “fastp: an ultra-fast all-in-one FASTQ preprocessor.” *Bioinformatics* 34: i884–i890. <https://doi.org/10.1093/bioinformatics/bty560>
6. Chen, Ying, Fan Nie, Shangqian Xie, Yingfeng Zheng, Qi Dai, Thomas Bray, Yaoxin Wang, et al. 2021. “Efficient assembly of nanopore reads via highly accurate and intact error correction.” *Nature Communications* 12: 60. <https://doi.org/10.1038/s41467-020-20236-7>
7. Vaser, Robert, Ivan Sović, Niranjan Nagarajan, and Mile Šikić. 2017. “Fast and accurate *de novo* genome assembly from long uncorrected reads.” *Genome Research* 27(5): 737–746. <https://doi.org/10.1101/gr.214270.116>
8. Walker, Bruce J, Thomas Abeel, Terrance Shea, Margaret Priest, Amr Abouelliel,Sharadha Sakthikumar, Christina A. Cuomo, et al. 2014. “Pilon: an integrated tool for comprehensive microbial variant detection and genome assembly improvement.” *PLoS ONE* 9(11): e112963. <https://doi.org/10.1371/journal.pone.0112963>
9. Servant, Nicolas, Nelle Varoquaux, Bryan R. Lajoie, Eric Viara, Chongjian Chen, Jean-Philippe Vert, Edith Heard, Job Dekker, and Emmanuel Barillot. 2015. “HiC-Pro: an optimized and flexible pipeline for Hi-C data processing.” *Genome Biology* 16: 259. <https://doi.org/10.1186/s13059-015-0831-x>
10. Dudchenko, Olga, Sanjit S. Batra, Arina D. Omer, Sarah K. Nyquist, Marie Hoeger, Neva C. Durand, Muhammad S. Shamim, et al. 2017. “*De novo* assembly of the *Aedes aegypti* genome using Hi-C yields chromosome-length scaffolds.” *Science* 356: 92–95. <https://doi.org/10.1126/science.aal3327>
11. Durand, Neva C, James T Robinson, Muhammad S Shamim, Ido Machol, Jill P Mesirov, Eric S Lander, and Erez Lieberman Aiden. 2016. “Juicebox provides a visualization system for Hi-C contact maps with unlimited zoom.” *Cell Systems* 3(1): 99–101. <https://doi.org/10.1016/j.cels.2015.07.012>
12. Li, Heng, Richard Durbin. 2009. “Fast and accurate short read alignment with Burrows–Wheeler transform.” *Bioinformatics* 25(4): 1754–1760. <https://doi.org/10.1093/bioinformatics/btp324>
13. Simão, Felipe A, Robert M. Waterhouse, Panagiotis Ioannidis, Evgenia V. Kriventseva, and Evgeny M. Zdobnov. 2015. “BUSCO: assessing genome assembly and annotation completeness with single-copy orthologs.” *Bioinformatics* 31(19): 3210–3212. <https://doi.org/10.1093/bioinformatics/btv351>
14. Xu, Zhao, Hao Wang. 2007. “LTR_FINDER: an efficient tool for the prediction of full-length LTR retrotransposons.” *Nucleic Acids Research* 35: W265–W268. <https://doi.org/10.1093/nar/gkm286>
15. Ellinghaus, David, Stefan Kurtz, and Ute Willhoeft. 2008. “LTRharvest, an efficient and flexible software for *de novo* detection of LTR retrotransposons.” *BMC Bioinformatics* 9: 18. <https://doi.org/10.1186/1471-2105-9-18>
16. Ou, Shujun, Ning Jiang. 2018. “LTR_retriever: a highly accurate and sensitive program for identification of long terminal repeat retrotransposons.” *Plant Physiology* 176(2): 1410–1422. <https://doi.org/10.1104/pp.17.01310>
17. Price, Alkes L, Neil C Jones, and Pavel A Pevzner. 2005. “*De novo* identification of repeat families in large genomes.” *Bioinformatics* 21: i351–i358. <https://doi.org/10.1093/bioinformatics/bti1018>
18. Bao, Weidong, Kenji K. Kojima, and Oleksiy Kohany. 2015. “Repbase Update, a database of repetitive elements in eukaryotic genomes.” *Mobile Dna* 6: 11. <https://doi.org/10.1186/s13100-015-0041-9>
19. Tempel, Sebastien. 2012. “Using and understanding RepeatMasker.” *Methods in Molecular Biology* 859: 29–51. <https://doi.org/10.1007/978-1-61779-603-6_2>
20. Benson, Gary. 1999. “Tandem repeats finder: a program to analyze DNA sequences.” *Nucleic Acids Research* 27(2): 573–580. <https://doi.org/10.1093/nar/27.2.573>
21. Stanke, Mario, Oliver Keller, Irfan Gunduz, Alec Hayes, Stephan Waack, and Burkhard Morgenstern. 2006. “AUGUSTUS: *ab initio* prediction of alternative transcripts.” *Nucleic Acids Research* 34: W435–W439. <https://doi.org/10.1093/nar/gkl200>
22. Korf, Ian. 2004. “Gene finding in novel genomes.” *BMC Bioinformatics* 5: 59. <https://doi.org/10.1186/1471-2105-5-59>
23. Aggarwal, Gautam, Ramakrishna Ramaswamy. 2002. “*Ab initio* gene identification: Prokaryote genome annotation with GeneScan and GLIMMER.” *Journal of Biosciences* 27: 7–14. <https://doi.org/10.1007/BF02703679>
24. Majoros, WH, M. Pertea, and S. L. Salzberg. 2004. “TigrScan and GlimmerHMM: two open source *ab initio* eukaryotic gene-finders.” *Bioinformatics* 20(16): 2878–2879. <https://doi.org/10.1093/bioinformatics/bth315>
25. Slater, Guy St C, and Ewan Birney. 2005. “Automated generation of heuristics for biological sequence comparison.” *BMC Bioinformatics* 6: 31. <https://doi.org/10.1186/1471-2105-6-31>
26. Pertea, Mihaela, Geo M Pertea, Corina M Antonescu, Tsung-Cheng Chang, Joshua T Mendell, and Steven L Salzberg. 2015. “StringTie enables improved reconstruction of a transcriptome from RNA-seq reads.” *Nature Biotechnology* 33(3): 290–295. <https://doi.org/10.1038/nbt.3122>
27. Haas, Brian J, Arthur L Delcher, Stephen M Mount, Jennifer R Wortman, Roger K Smith Jr, Linda I Hannick, Rama Maiti, et al. 2003. “Improving the *Arabidopsis* genome annotation using maximal transcript alignment assemblies.” *Nucleic Acids Research* 31(19): 5654–5666. <https://doi.org/10.1093/nar/gkg770>
28. Haas, Brian J, Steven L Salzberg, Wei Zhu, Mihaela Pertea, Jonathan E Allen, Joshua Orvis, Owen White, C Robin Buell, and Jennifer R Wortman. 2008. “Automated eukaryotic gene structure annotation using EVidenceModeler and the program to assemble spliced alignments.” *Genome Biology* 9: R7. <https://doi.org/10.1186/gb-2008-9-1-r7>
29. Mistry, Jaina, Robert D. Finn, Sean R. Eddy, Alex Bateman, and Marco Punta. 2013. “Challenges in homology search: HMMER3 and convergent evolution of coiled-coil regions.” *Nucleic Acids Research* 41: e121. <https://doi.org/10.1093/nar/gkt263>
30. Jones, Philip, David Binns, Hsin-Yu Chang, Matthew Fraser, Weizhong Li, Craig McAnulla, Hamish McWilliam, et al. 2014. “InterProScan 5: genome-scale protein function classification.” *Bioinformatics* 30(9): 1236–1240. <https://doi.org/10.1093/bioinformatics/btu031>
31. Lowe, Todd M, Sean R. Eddy. 1997. “tRNAscan-SE: a program for improved detection of transfer RNA genes in genomic sequence.” *Nucleic Acids Research* 25(5): 955–964. <https://doi.org/10.1093/nar/25.5.955>
32. Lagesen, Karin, Peter Hallin, Einar Andreas Rødland, Hans-Henrik Stærfeldt, Torbjorn Rognes, and David W Ussery. 2007. “RNAmmer: consistent and rapid annotation of ribosomal RNA genes.” *Nucleic Acids Research* 35(9): 3100–3108. <https://doi.org/10.1093/nar/gkm160>
33. Nawrocki, Eric P, Diana L. Kolbe, and Sean R. Eddy. 2009. “Infernal 1.0: inference of RNA alignments.” *Bioinformatics* 25(10): 1335–1337. <https://doi.org/10.1093/bioinformatics/btp157>
34. Griffiths-Jones, Sam, Simon Moxon, Mhairi Marshall, Ajay Khanna, Sean R. Eddy, and Alex Bateman. 2005. “Rfam: annotating non-coding RNAs in complete genomes.” *Nucleic Acids Research* 33: D121–D124. <https://doi.org/10.1093/nar/gki081>
35. Zapata, Luis, Jia Ding, Eva-Maria Willing, Benjamin Hartwig, Daniela Bezdan, Wenbiao Jiao, Vipul Patel, et al. 2016. “Chromosome-level assembly of *Arabidopsis thaliana* L*er* reveals the extent of translocation and inversion polymorphisms.” *Proceedings of the National Academy of Sciences* 113(28): E4052–E4060. <https://doi.org/10.1073/pnas.1607532113>
36. Jaillon, Olivier, Jean-Marc Aury, Benjamin Noel, Alberto Policriti, Christian Clepet, Alberto Casagrande, Nathalie Choisne, et al. 2007. “The grapevine genome sequence suggests ancestral hexaploidization in major angiosperm phyla.” *Nature* 449: 463–467. <https://doi.org/10.1038/nature06148>
37. Raymond, Olivier, Jerome Gouzy, Jeremy Just, Helene Badouin, Marion Verdenaud, Arnaud Lemainque, Philippe Vergne, et al. 2018. “The *Rosa* genome provides new insights into the domestication of modern roses.” *Nature Genetics* 50(6): 772–777. <https://doi.org/10.1038/s41588-018-0110-3>
38. Tan, Qiuping, Sen Li, Yuzheng Zhang, Min Chen, Binbin Wen, Shan Jiang, Xiude Chen, et al. 2021. “Chromosome-level genome assemblies of five *Prunus* species and genome-wide association studies for key agronomic traits in peach.” *Horticulture Research* 8: 213. <https://doi.org/10.1038/s41438-021-00648-2>
39. Alioto, Tyler, Konstantinos G. Alexiou, Amélie Bardil, Fabio Barteri, Raúl Castanera, Fernando Cruz, Amit Dhingra, et al. 2020. Transposons played a major role in the diversification between the closely related almond and peach genomes: results from the almond genome sequence.” *The Plant Journal* 101(2): 455–472. <https://doi.org/10.1111/tpj.14538>
40. Groppi, Alexis, Shuo Liu, Amandine Cornille, Stephane Decroocq, Quynh Trang Bui, David Tricon, Corinne Cruaud, et al. 2021. “Population genomics of apricots unravels domestication history and adaptive events.” *Nature Communications* 12: 3956. <https://doi.org/10.1038/s41467-021-24283-6>
41. Zheng, Tangchun, Ping Li, Xiaokang Zhuo, Weichao Liu, Like Qiu, Lulu Li, Cunquan Yuan, et al. 2022. “The chromosome‐level genome provides insight into the molecular mechanism underlying the tortuous‐branch phenotype of *Prunus mume*.” *New Phytologist* 235(1): 141–156. <https://doi.org/10.1111/nph.17894>
42. Wang, Jiawei, Weizhen Liu, Dongzi Zhu, Po Hong, Shizhong Zhang, Shijun Xiao, Yue Tan, et al. 2020. “Chromosome-scale genome assembly of sweet cherry (*Prunus avium* L.) cv. Tieton obtained using long-read and Hi-C sequencing.” *Horticulture Research* 7: 122. https://doi.org/10.1038/s41438-020-00343-8
43. Goeckeritz, Charity Z, Kathleen E Rhoades, Kevin L Childs, Amy F Iezzoni, Robert VanBuren, and Courtney A Hollender. 2023. “Genome of tetraploid sour cherry (*Prunus cerasus* L*.*) ‘Montmorency’ identifies three distinct ancestral *Prunus* genomes.” *Horticulture Research* 10(7): uhad097. <https://doi.org/10.1093/hr/uhad097>
44. Wöhner, Thomas W., Ofere F Emeriewen, Alexander H J Wittenberg, Harrie Schneiders, Ilse Vrijenhoek, Julia Halász, Karoly Hrotkó, et al. 2021. “The draft chromosome-level genome assembly of tetraploid ground cherry (*Prunus fruticosa* Pall*.*) from long reads.” *Genomics* 113(6): 4173–4183. <https://doi.org/10.1016/j.ygeno.2021.11.002>
45. Nie, Chaoren, Yingjie Zhang, Xiaoqin Zhang, Wensheng Xia, Hongbing Sun, Sisi Zhang, Na Li, et al. 2023. “Genome assembly, resequencing and genome‐wide association analyses provide novel insights into the origin, evolution and flower colour variations of flowering cherry.” *The Plant Journal* 114(3): 519–533. <https://doi.org/10.1111/tpj.16151>
46. Jiu, Songtao, Baozheng Chen, Xiao Dong, Zhengxin Lv, Yuxuan Wang, Chunjin Yin, Yan Xu, et al. 2023. “Chromosome-scale genome assembly of *Prunus pusilliflora* provides novel insights into genome evolution, disease resistance, and dormancy release in *Cerasus* L.” *Horticulture Research* 10(5): uhad062. <https://doi.org/10.1093/hr/uhad062>
47. Yi, Xiangui, Xiaqing Yu, Jie Chen, Min Zhang, Shaowei Liu, Hong Zhu, Meng Li et al. 2020. “The genome of Chinese flowering cherry (*Cerasus serrulata*) provides new insights into *Cerasus* species.” *Horticulture Research* 7: 165. <https://doi.org/10.1038/s41438-020-00382-1>
48. Baek, Seunghoon, Kyung Choi, Goon-Bo Kim, Hee-Ju Yu, Aea Cho, Hoyeol Jang, Changkyun Kim, et al. 2018. “Draft genome sequence of wild *Prunus yedoensis* reveals massive inter-specific hybridization between sympatric flowering cherries.” *Genome Biology* 19: 127. <https://doi.org/10.1186/s13059-018-1497-y>
49. Edgar, Robert C. 2004. MUSCLE: multiple sequence alignment with high accuracy and high throughput.” *Nucleic Acids Research* 32(5): 1792–1797. <https://doi.org/10.1093/nar/gkh340>
50. Yano, Kenji, Eiji Yamamoto, Koichiro Aya, Hideyuki Takeuchi, Pei-ching Lo, Li Hu, Masanori Yamasaki, et al. 2016. “Genome-wide association study using whole-genome sequencing rapidly identifies new genes influencing agronomic traits in rice.” *Nature Genetics* 48: 927–934. <https://doi.org/10.1038/ng.3596>
51. De, Bie Tijl, Nello Cristianini, Jeffery P. Demuth, and Matthew W. Hahn. 2006. “CAFE: a computational tool for the study of gene family evolution.” *Bioinformatics* 22(10): 1269–1271. <https://doi.org/10.1093/bioinformatics/btl097>
52. Goel, Manish, Hequan Sun, Wen-Biao Jiao, and Korbinian Schneeberger, 2019. “SyRI: finding genomic rearrangements and local sequence differences from whole-genome assemblies.” *Genome Biology* 20: 277. <https://doi.org/10.1186/s13059-019-1911-0>
53. Sun, Pengchuan, Beibei Jiao, Yongzhi Yang, Lanxing Shan, Ting Li, Xiaonan Li, Zhenxiang Xi, Xiyin Wang, and Jianquan Liu. 2022. “WGDI: A user-friendly toolkit for evolutionary analyses of whole-genome duplications and ancestral karyotypes.” *Molecular Plant* 15(12): 1841–1851. <https://doi.org/10.1016/j.molp.2022.10.018>
54. Tang, Haibao, John E Bowers, Xiyin Wang, Ray Ming, Maqsudul Alam, and Andrew H Paterson. 2008. “Synteny and collinearity in plant genomes.” *Science* 320(5875): 486–488. <https://doi.org/10.1126/science.1153917>
55. Suyama, Mikita, David Torrents, and Peer Bork. 2006. “PAL2NAL: Robust conversion of protein sequence alignments into the corresponding codon alignments.” *Nucleic Acids Research* 34: W609–W612. <https://doi.org/10.1093/nar/gkl315>
56. Ziheng, Yang. 2007. “PAML 4: Phylogenetic analysis by maximum likelihood.” *Molecular Biology and Evolution* 24(8): 1586–1591. <https://doi.org/10.1093/molbev/msm088>
57. Graphodatsky, Alexander S, Vladimir A Trifonov, and Roscoe Stanyon. 2011. “The genome diversity and karyotype evolution of mammals.” *Molecular Cytogenetics* 4(1): 22. <https://doi.org/10.1186/1755-8166-4-22>
58. Hill, Jason, Pasi Rastas, Emily A. Hornett, Ramprasad Neethiraj, Nathan Clark, Nathan Morehouse, Maria de la Paz Celorio-Mancera, et al. 2019**.** “Unprecedented reorganization of holocentric chromosomes provides insights into the enigma of lepidopteran chromosome evolution.” *Science Advances* 5(6): eaat3648. <https://doi.org/10.1126/sciadv.aau3648>
59. Yang, Haiying, Changping Tian, Shujun Ji, Fengzhu Ni, Xinguang Fan, Yanqing Yang, Chanchan Sun, Hansheng Gong, and Aidi Zhang. 2021. “Integrative analyses of metabolome and transcriptome reveals metabolomic variations and candidate genes involved in sweet cherry (*Prunus avium L.*) fruit quality during development and ripening.” *PLoS ONE* 16(11): e0260004. <https://doi.org/10.1371/journal.pone.0260004>
60. Pertea, Mihaela, Daehwan Kim, Geo M Pertea, Jeffrey T Leek, and Steven L Salzberg. 2016. “Transcript-level expression analysis of RNA-seq experiments with HISAT, StringTie and Ballgown.” *Nature Protocols* 11(9): 1650–1667. <https://doi.org/10.1038/nprot.2016.095>
61. Love, Michael I, Wolfgang Huber, and Simon Anders. 2014. “Moderated estimation of fold change and dispersion for RNA-seq data with DESeq2.” *Genome Biology* 15(12): 550. <https://doi.org/10.1186/s13059-014-0550-8>
62. Artati, Anna, Cornelia Prehn, and Jerzy Adamski. 2019. “LC–MS/MS-based metabolomics for cell cultures.” *Methods in Molecular Biology* 1994: 119–130. <https://doi.org/10.1007/978-1-4939-9477-9_10>
63. Wang, Yuxuan, Yan Xu, Jieming Xu, Wanxia Sun, Zhengxin Lv, Muhammad Aamir Manzoor, Xunju Liu, et al. 2023. “Oxygenation alleviates waterlogging-caused damages to cherry rootstocks.” *Molecular Horticulture* 3: 8. <https://doi.org/10.1186/s43897-023-00056-1>
64. Yan, Xu, Zhengxin Lv, Muhammad Aamir Manzoor, Linhong Song, Maosen Wang, Lei Wang, Shiping Wang, et al. 2024. “VvD14c–VvMAX2–VvLOB/VvLBD19 module is involved in the strigolactone-mediated regulation of grapevine root architecture.” *Molecular Horticulture* 4(1): 40. <https://doi.org/10.1186/s43897-024-00117-z>
65. Livak, Kenneth J, Thomas D. Schmittgen. 2001. “Analysis of relative gene expression data using real-time quantitative PCR and the 2^−ΔΔC^_T_ Method.” *Methods* 25(4): 402–408. <https://doi.org/10.1006/meth.2001.1262>
66. Jiu, Songtao, Yan Xu, Jiyuan Wang, Lei Wang, Shiping Wang, Chao Ma, Le Guan, et al. 2019. “Genome-wide identification, characterization, and transcript analysis of the TCP transcription factors in *Vitis vinifera*.” *Frontiers in Genetics* 10: 1276. <https://doi.org/10.3389/fgene.2019.01276>
67. Jiu, Songtao, Le Guan, Xiangpeng Leng, Kekun Zhang, Salman Haider M, Xiang Yu, Xudong Zhu, et al. 2021. “The role of *VvMYBA2r* and *VvMYBA2w* alleles of *MYBA2* locus in the regulation of anthocyanin biosynthesis for molecular breeding of grape (*Vitis* spp.) skin coloration.” P*lant Biotechnology Journal* 19(6): 1216–1239. <https://doi.org/10.1111/pbi.13543>


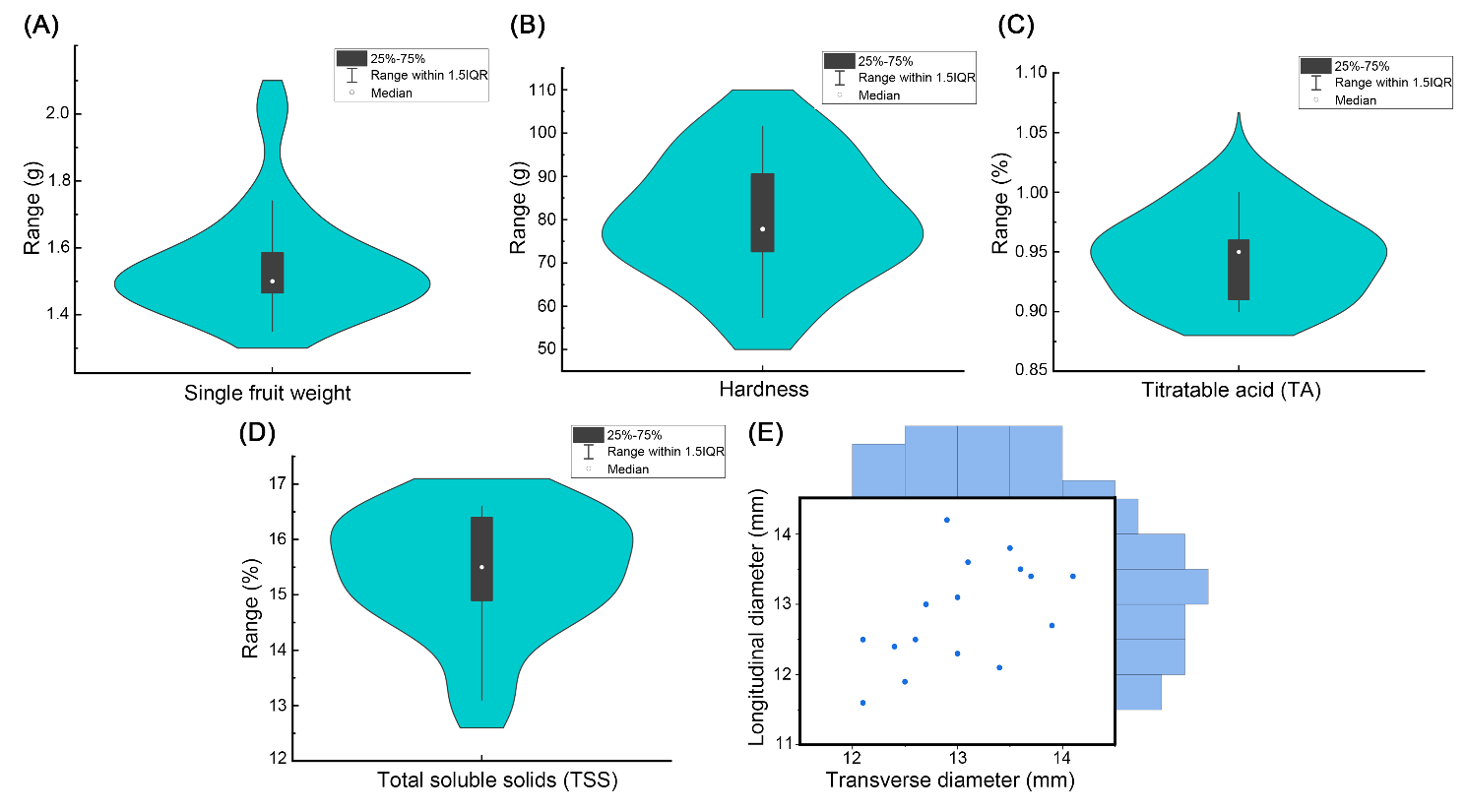


**Figure S1** *P. tomentosa* fruit parameters, including (A) single fruit weight, (B) hardness, (C) titratable acid, (D) total soluble solids, and (E) longitudinal and transverse diameters of fruits.


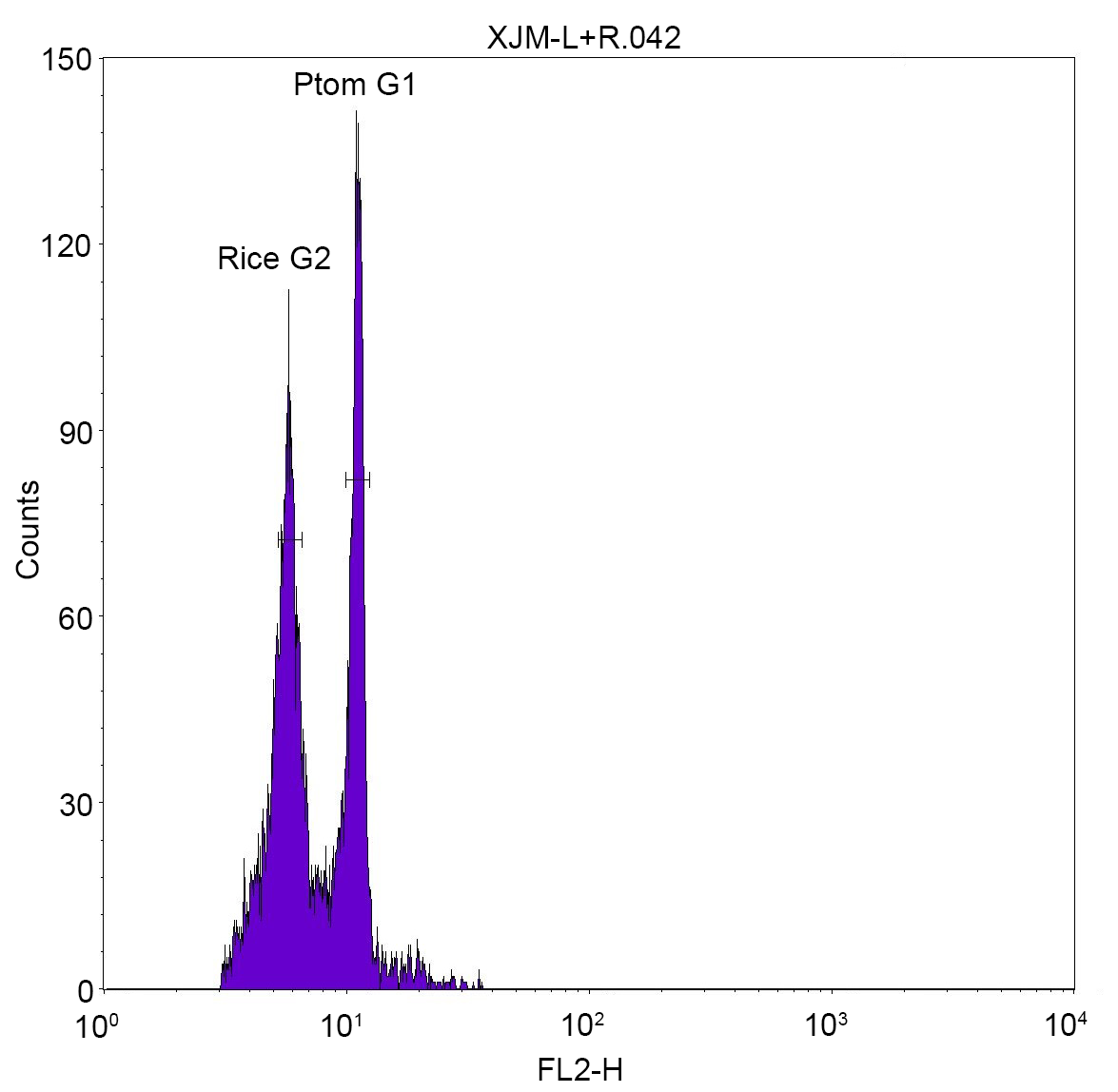


**Figure S2** Estimation of *Prunus tomentosa* genome size*.* PI positive gated population in a histogram illustrating PI-stained *P. tomentosa* (Ptom) and *Oryza sativa* (Rice). PI, propidium iodide.


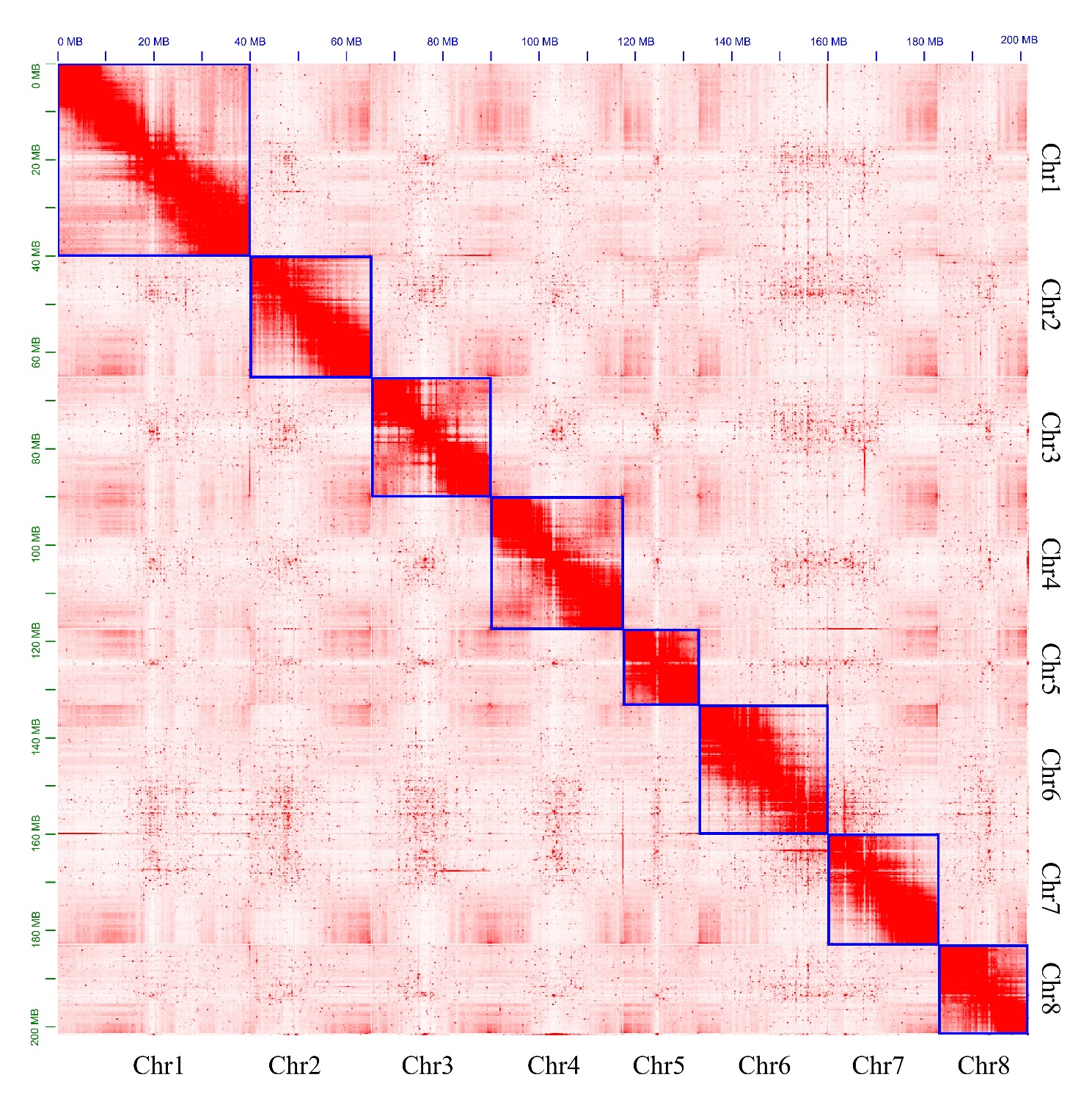


**Figure S3** High-resolution Hi-C contact matrix in the chromosome-level assembly of the *Prunus tomentosa* genome. Individual Chrs were scaffolded and independently assembled.


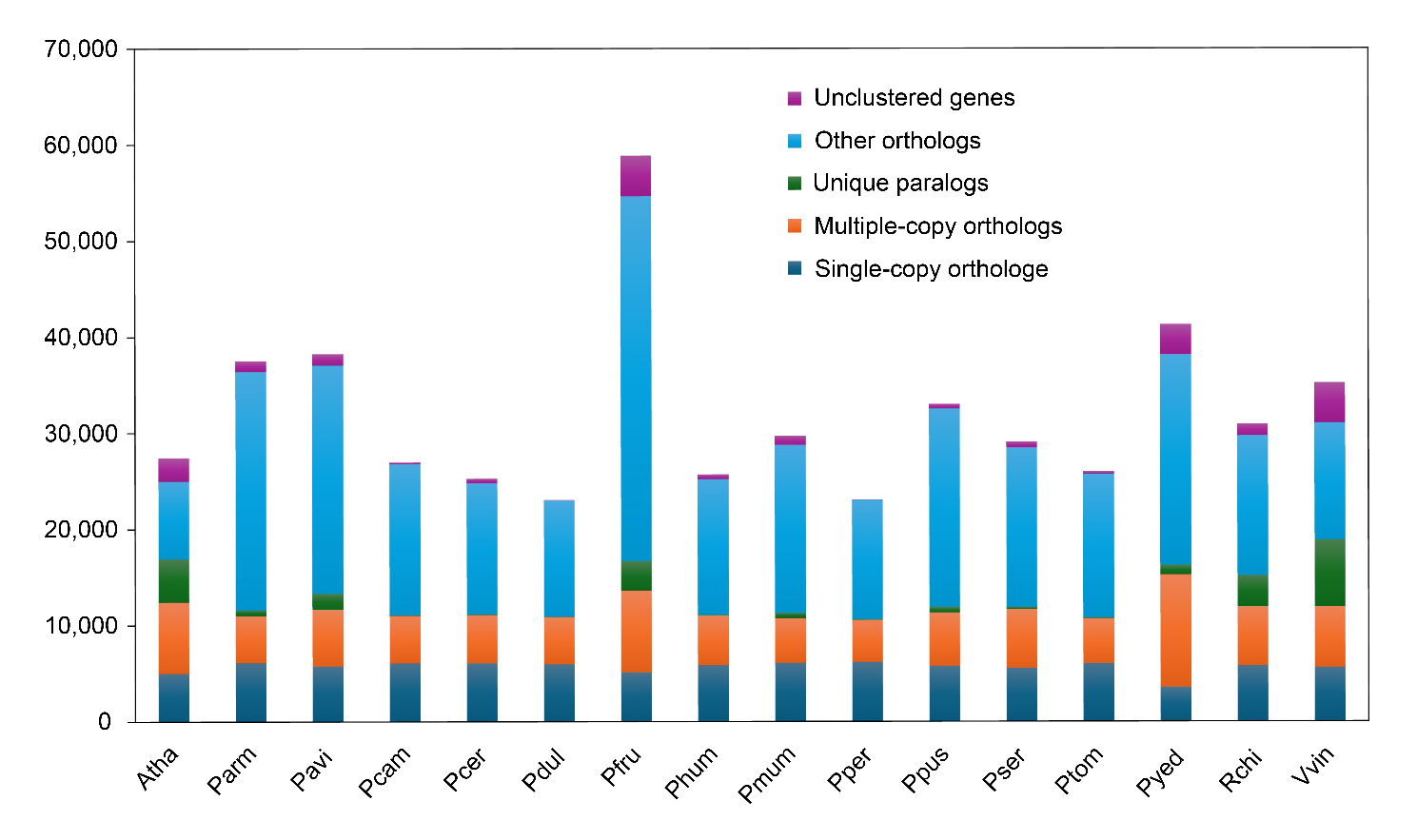


**Figure S4** Gene number distribution of single- and multiple copies and other orthologs, including unique paralogs and unclustered genes in *Arabidopsis thaliana* (Atha), *Vitis vinifera* (Vvin), *Rosa chinensis* (Rchi), *Prunus persica* (Pper), *Prunus dulcis* (Pdul), *Prunus armeniaca* (Parm), *Prunus mume* (Pmum), *Prunus humilis* (Phum), *Prunus avium* (Pavi), *Prunus cerasus* (Pcer), *Prunus fruticosa* (Pfru), *Prunus campanulata* (Pcam), *Prunus pusilliflora* (Ppus), *Prunus serrulata* (Pser), *Prunus yedoensis* (Pyed), and *P. tomentosa* (Ptom).


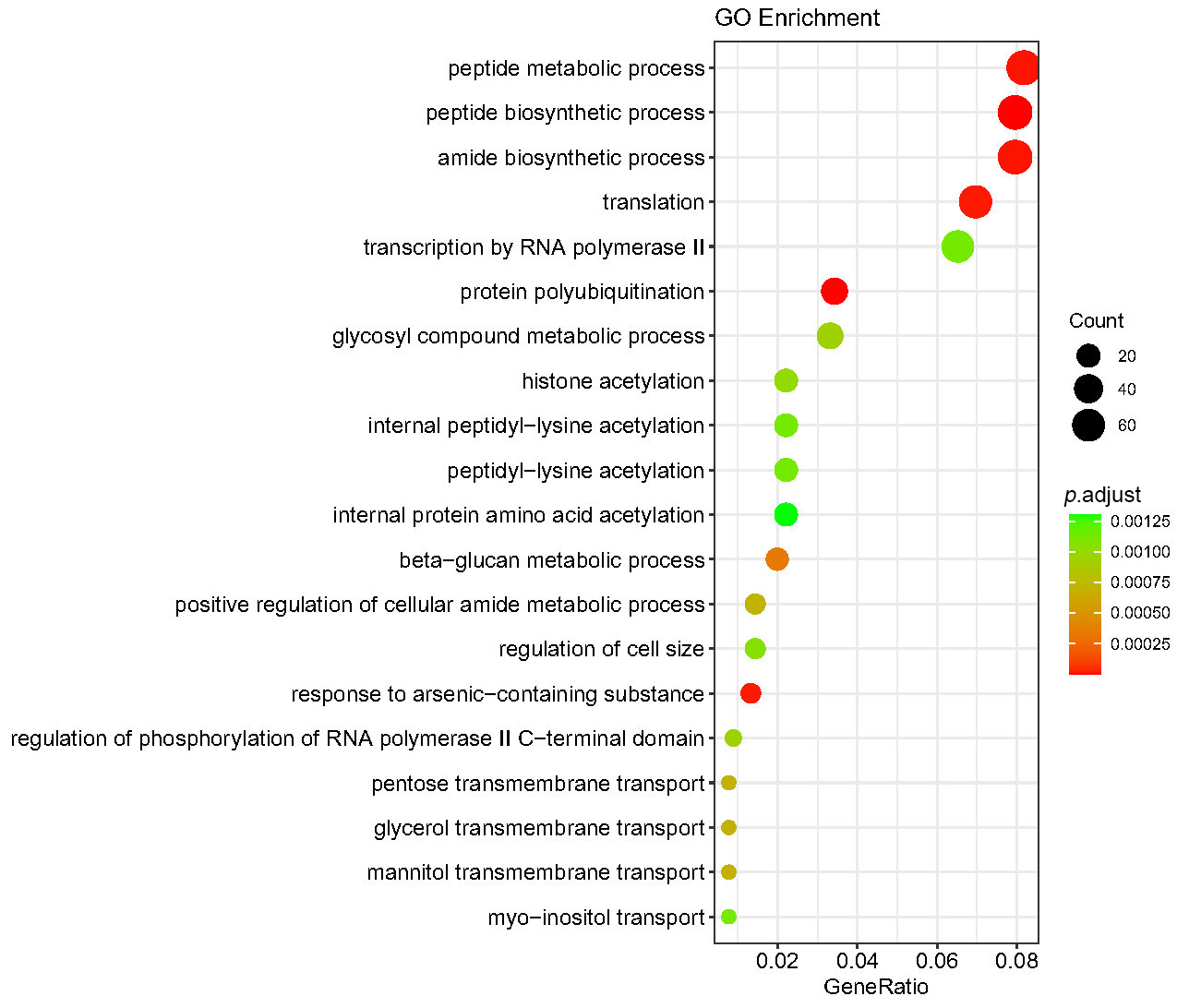


**Figure S5** GO enrichment analysis for the expanded gene families in *Prunus tomentosa.* GO, Gene Ontology.


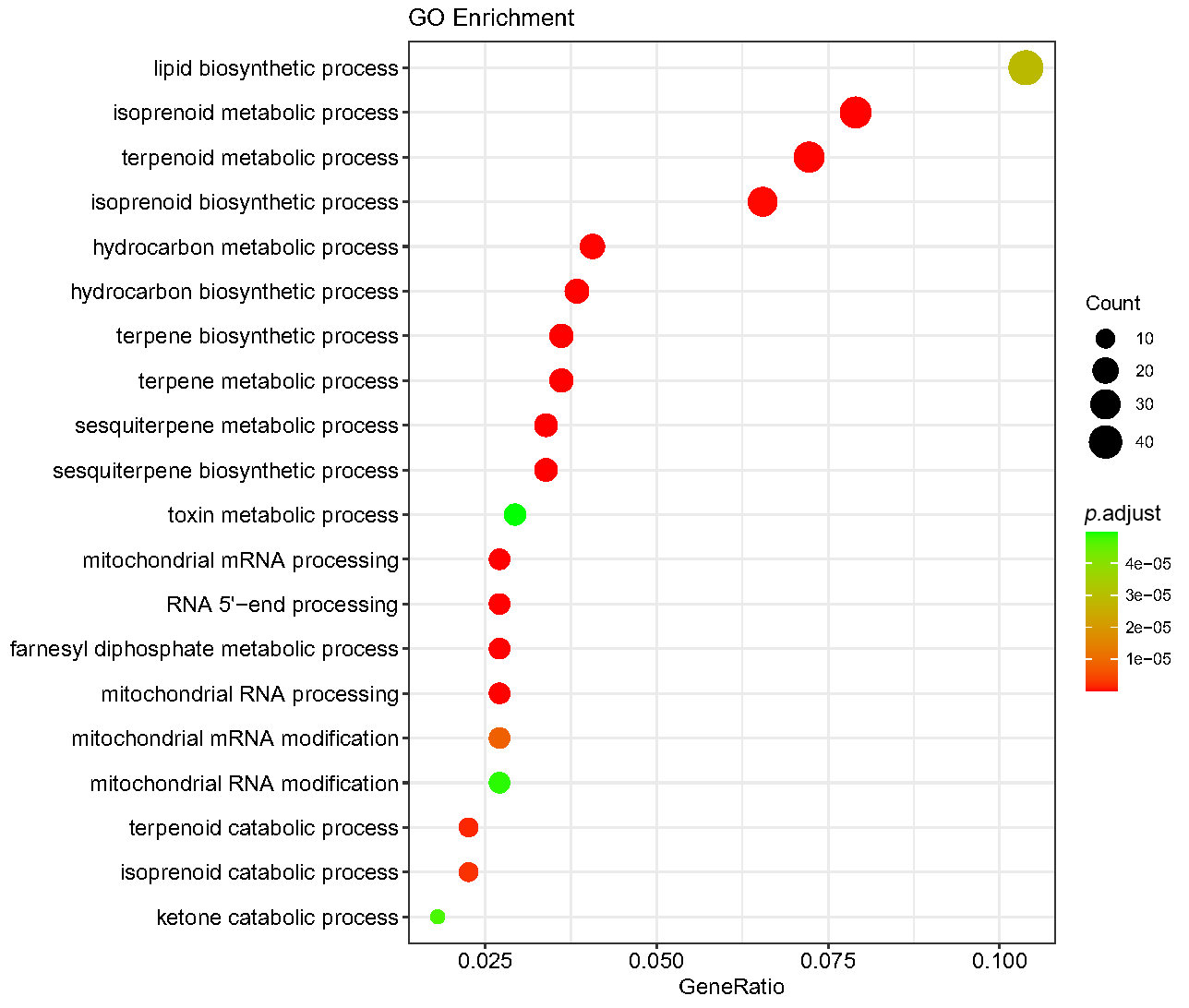


**Figure S6** GO enrichment analysis for the contracted gene families in *Prunus tomentosa.* GO, Gene Ontology.

**
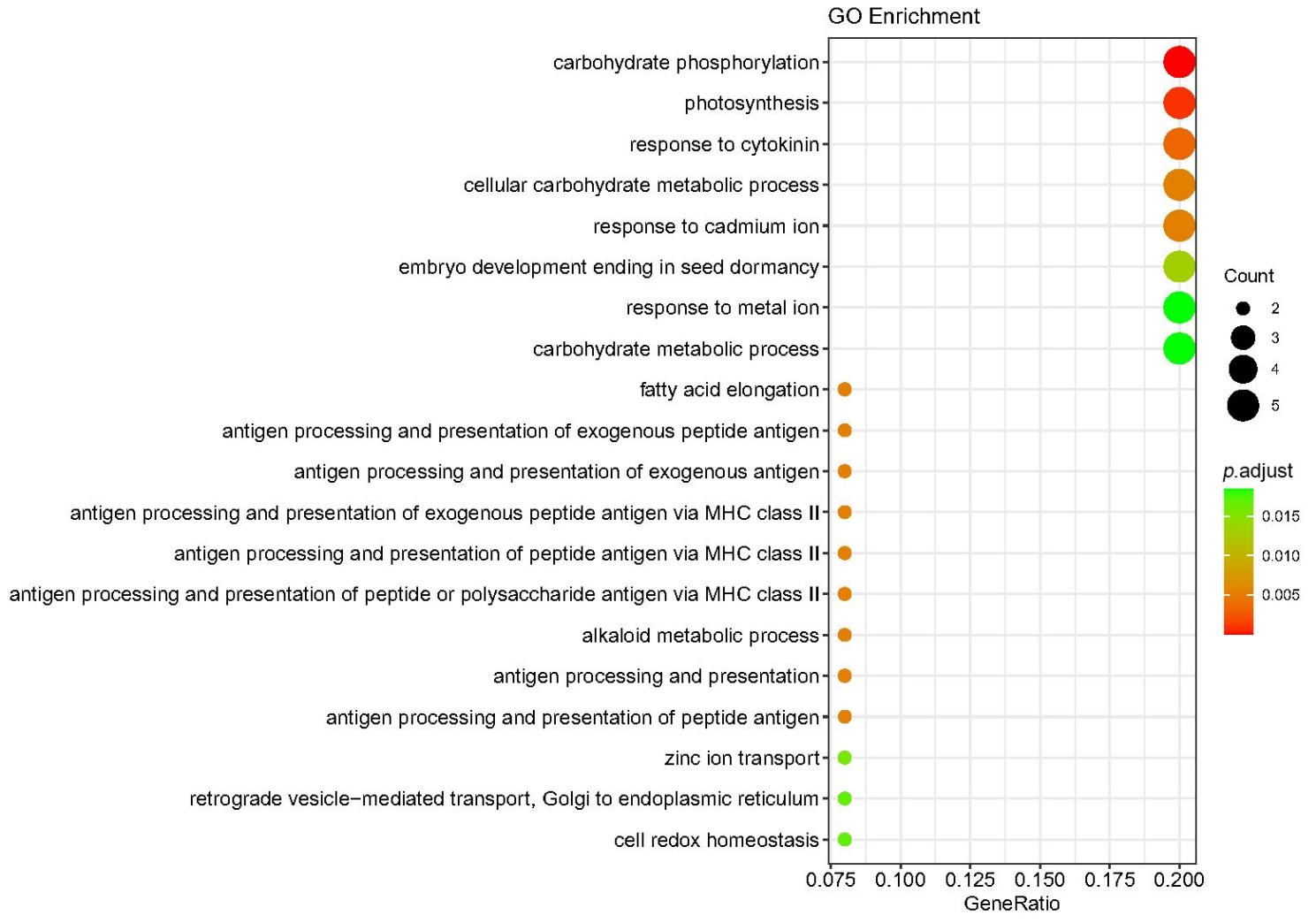
**

**Figure S7** GO enrichment analysis for the unique gene families in *Prunus tomentosa.* GO, Gene Ontology.


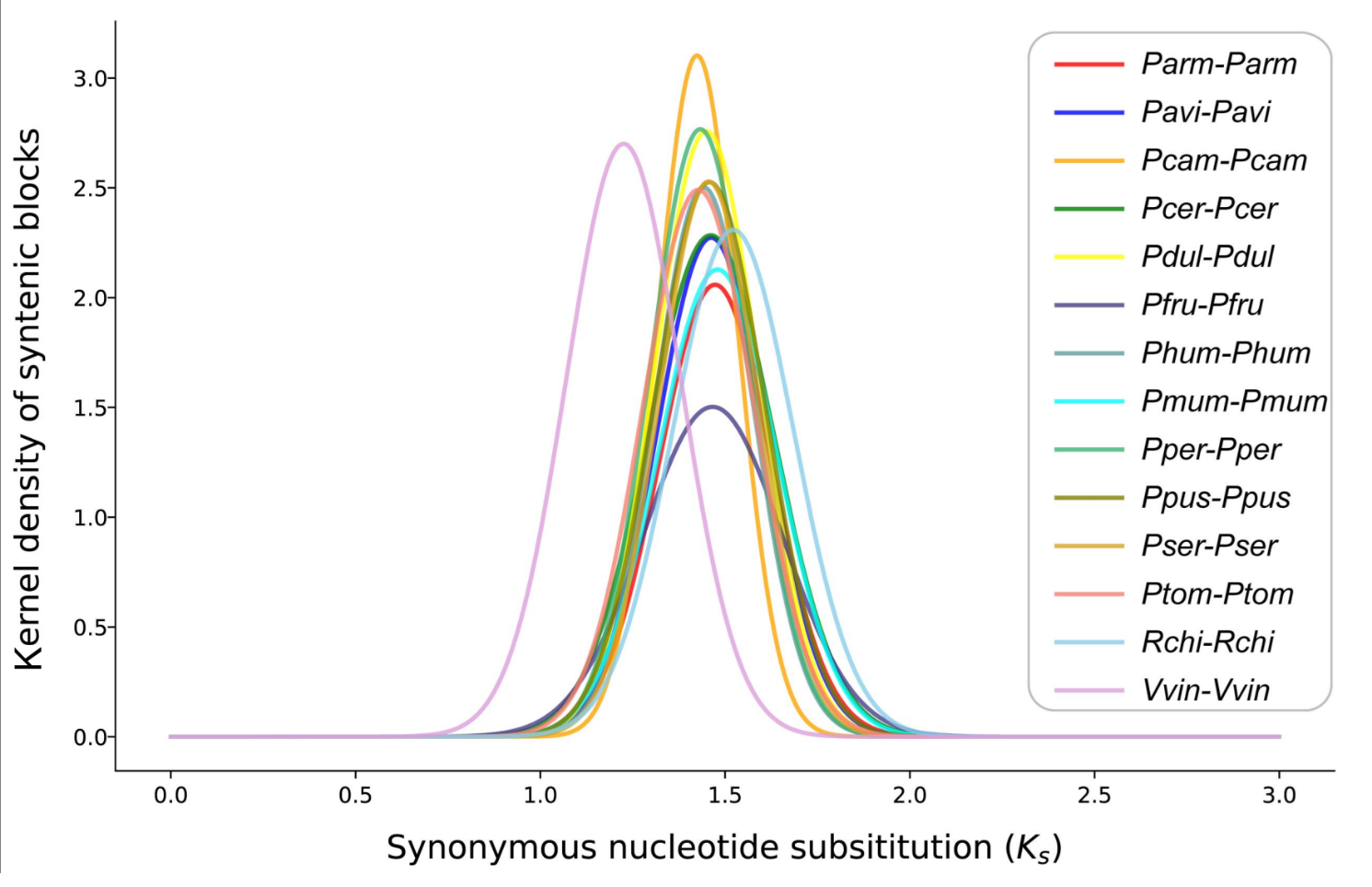


**Figure S8** *Ks* density curve before correction. Solid lines of different colors represent the Ks density distribution of different species.


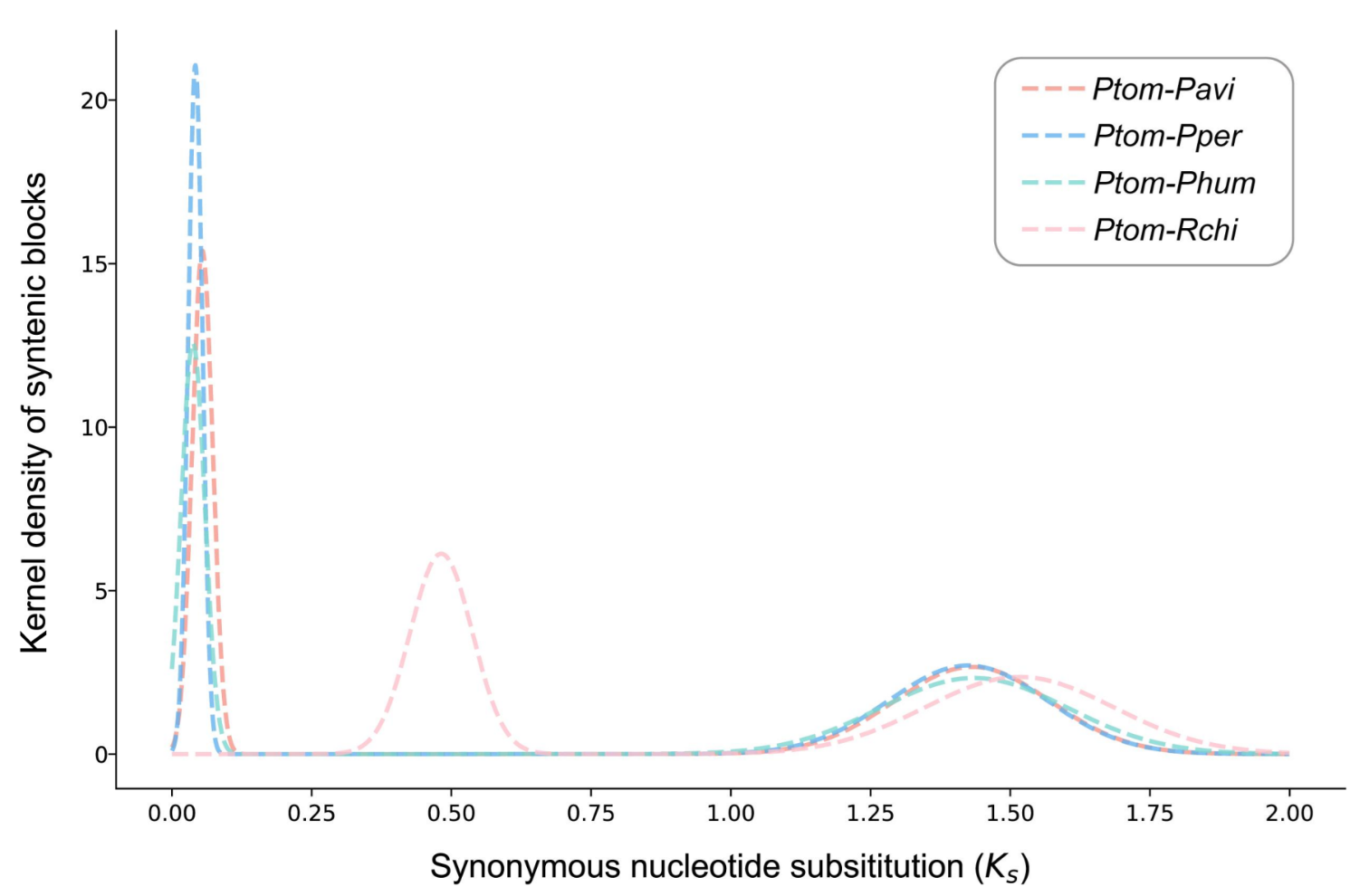


**Figure S9** Distribution map of *Ks* density between species. Dashed lines of different colors represent the distributions of *Ks* density for different species.


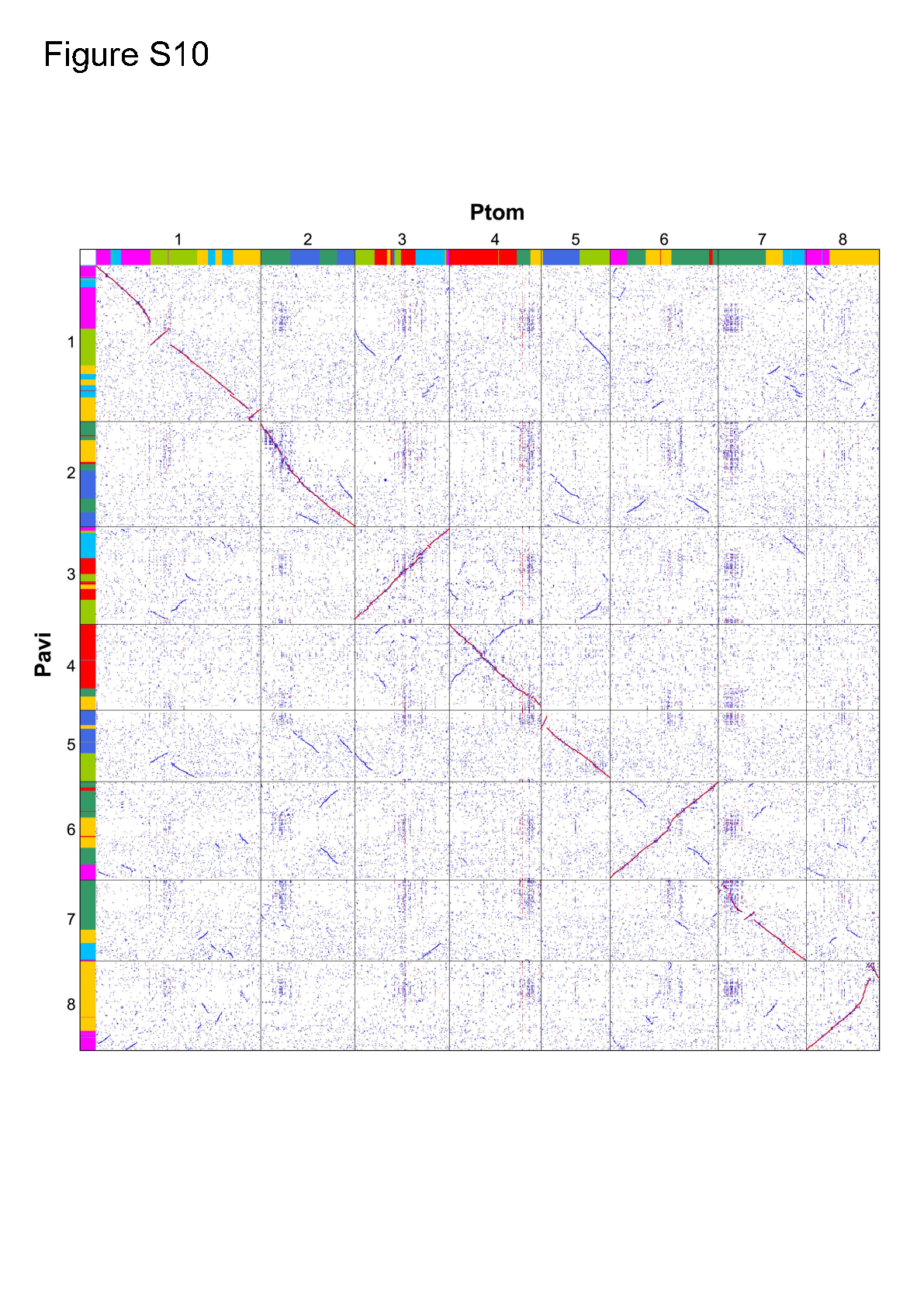


**Figure S10** Homologous dotplots between *Prunus avium* and *Prunus tomentosa*. The numbers in the figure represent the chromosome numbers of each species. Red fragments represent species with the best match, and blue segments indicate the second-best match. Red dotted line segments do not overlap in the horizontal and vertical directions, indicating that the ratio of the best-matched homologous regions was 1:1. The colors on chromosomes represent the colors of seven chromosomes in the ancestral eudicots karyotype.


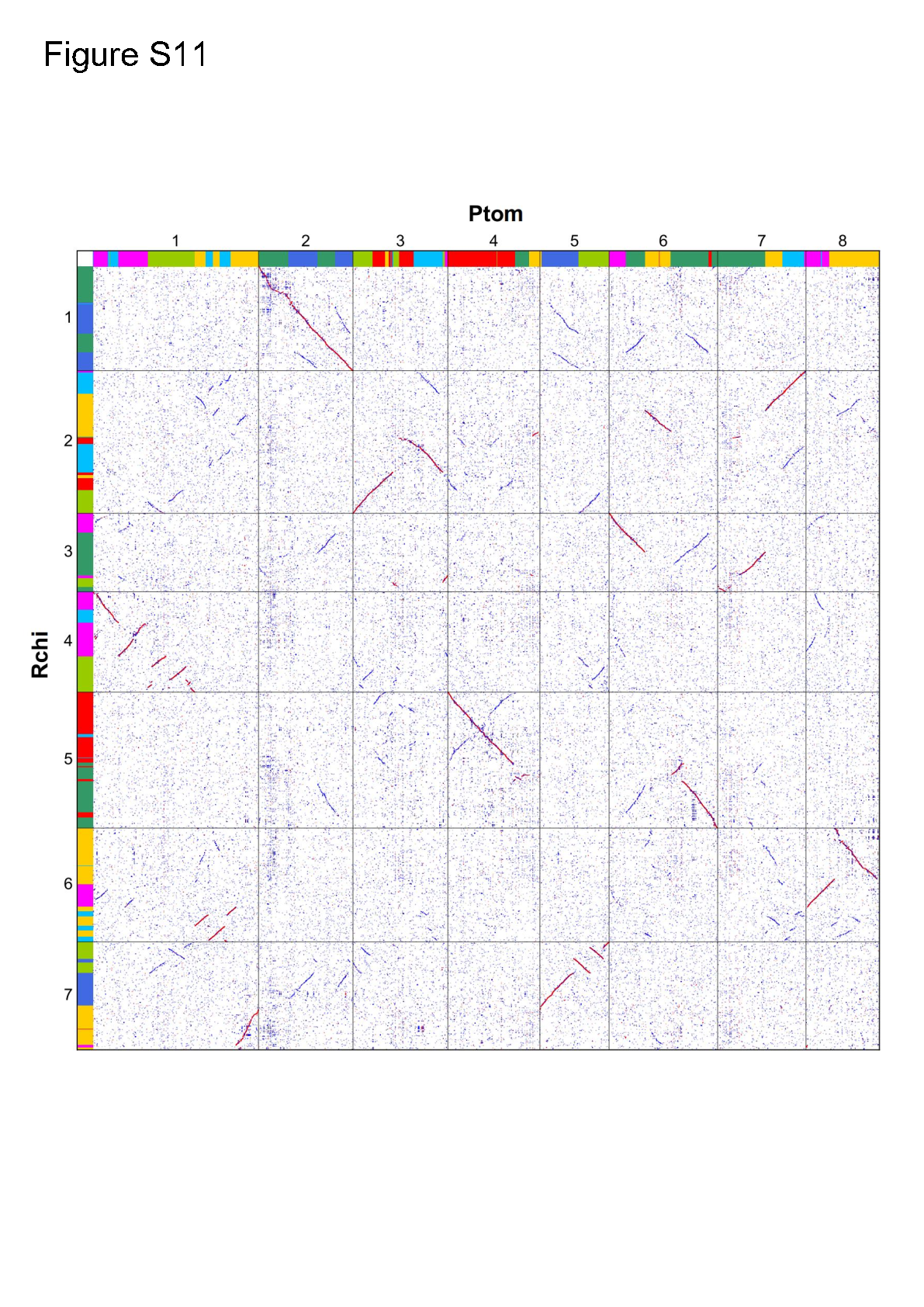


**Figure S11** Homologous dotplots between *Rosa chinensis* and *Prunus tomentosa*. The numbers in the figure represent the chromosome numbers of each species. Red fragments represent species with the best match, and blue segments indicate the second-best match. The line segments of red dots do not overlap in the horizontal and vertical directions, suggesting that the ratio of the best-matched homologous regions was 1:1. The colors on chromosomes represent the colors of seven chromosomes in the ancestral eudicot karyotype.


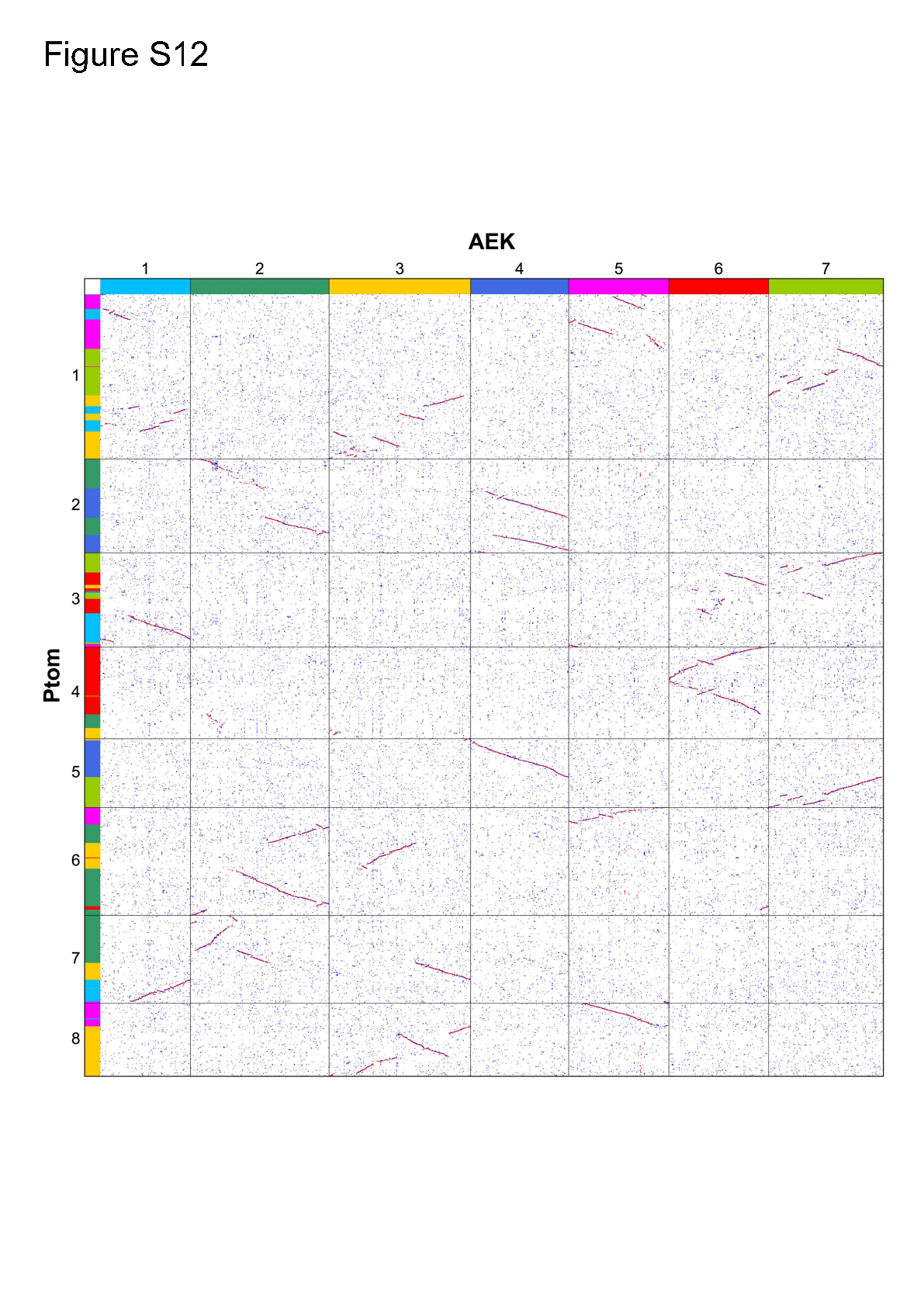


**Figure S12** Homologous dotplots between *Prunus tomentosa* and ancestors of core eudicots. The numbers in the figure represent the chromosome numbers of each species. Red fragments represent species with the best match, and blue segments indicate the second-best match. The red dotted line segments do not overlap in the horizontal and vertical directions, suggesting that the ratio of the best-matched homologous regions was 1:1. The colors on chromosomes represent the colors of seven chromosomes in the ancestral eudicots karyotype.
